# Differential effects of two-hit models of acute and ventilator-induced lung injury on lung structure, function, and inflammation

**DOI:** 10.3389/fphys.2023.1217183

**Published:** 2023-07-26

**Authors:** Jill Bilodeaux, Huda Farooqi, Maria Osovskaya, Alexander Sosa, Alison Wallbank, Lars Knudsen, Peter D. Sottile, David J. Albers, Bradford J. Smith

**Affiliations:** ^1^ Department of Bioengineering, University of Colorado Denver | Anschutz Medical Campus, Aurora, CO, United States; ^2^ Department of Microbiology, University of Colorado Denver/Anschutz Medical Campus, Aurora, Germany; ^3^ Institute of Functional and Applied Anatomy, Hannover Medical School, Hannover, Germany; ^4^ Biomedical Research in Endstage and Obstructive Lung Disease Hannover (BREATH), Member of the German Centre for Lung Research (DZL), Hannover, Germany; ^5^ Division of Pulmonary Sciences and Critical Care Medicine, Department of Medicine, University of Colorado School of Medicine, Aurora, CO, United States; ^6^ Section of Informatics and Data Science, Department of Pediatrics, School of Medicine, University of Colorado Denver, Anschutz Medical Campus, Aurora, CO, United States; ^7^ Section of Pulmonary and Sleep Medicine, Department of Pediatrics, University of Colorado Denver, Anschutz Medical Campus, Aurora, CO, United States

**Keywords:** acute lung injury, mechanical ventilation, ventilator-induced lung injury, morphometry, stereology

## Abstract

Acute respiratory distress syndrome (ARDS) and acute lung injury have a diverse spectrum of causative factors including sepsis, aspiration of gastric contents, and near drowning. Clinical management of severe lung injury typically includes mechanical ventilation to maintain gas exchange which can lead to ventilator-induced lung injury (VILI). The cause of respiratory failure is acknowledged to affect the degree of lung inflammation, changes in lung structure, and the mechanical function of the injured lung. However, these differential effects of injury and the role of etiology in the structure-function relationship are not fully understood. To address this knowledge gap we caused lung injury with intratracheal hydrochloric acid (HCL) or endotoxin (LPS) 2 days prior to ventilation or with an injurious lavage (LAV) immediately prior to ventilation. These injury groups were then ventilated with high inspiratory pressures and positive end expiratory pressure (PEEP) = 0 cmH_2_O to cause VILI and model the clinical course of ARDS followed by supportive ventilation. The effects of injury were quantified using invasive lung function measurements recorded during PEEP ladders where the end-expiratory pressure was increased from 0 to 15 cm H_2_O and decreased back to 0 cmH_2_O in steps of 3 cmH_2_O. Design-based stereology was used to quantify the parenchymal structure of lungs air-inflated to 2, 5, and 10 cmH_2_O. Pro-inflammatory gene expression was measured with real-time quantitative polymerase chain reaction and alveolocapillary leak was estimated by measuring bronchoalveolar lavage protein content. The LAV group had small, stiff lungs that were recruitable at higher pressures, but did not demonstrate substantial inflammation. The LPS group showed septal swelling and high pro-inflammatory gene expression that was exacerbated by VILI. Despite widespread alveolar collapse, elastance in LPS was only modestly elevated above healthy mice (CTL) and there was no evidence of recruitability. The HCL group showed increased elastance and some recruitability, although to a lesser degree than LAV. Pro-inflammatory gene expression was elevated, but less than LPS, and the airspace dimensions were reduced. Taken together, those data highlight how different modes of injury, in combination with a 2^nd^ hit of VILI, yield markedly different effects.

## 1 Introduction

Acute Respiratory Distress Syndrome (ARDS) is caused by factors ranging from aspiration of gastric contents to sepsis with more than ≈200,000 cases per typical year in the United States (Rubenfeld et al., 2005) and an estimated mortality rate of ≈35–45%, with mortality increasing with severity (Bellani et al., 2016). The COVID-19 pandemic caused a dramatic increase in ARDS and brought this syndrome into public focus. Independent of etiology, ARDS patients require life-sustaining mechanical ventilation support to overcome impaired gas exchange and derangements in lung function caused by pulmonary edema, surfactant inactivation, and alveolar collapse (Swenson and Swenson, 2021).

Although mechanical ventilation is a necessary, lifesaving intervention for ARDS, it is not without consequences. Prolonged mechanical ventilation can cause ventilator-induced lung injury (VILI) through tissue overdistension (volutrauma), the cyclic collapse and reopening of small airways and alveoli (atelectrauma), and secondary inflammatory effects (biotrauma) (Bates and Smith, 2018). These additional injuries conspire to increase ARDS morbidity and mortality. To reduce VILI, lung protective ventilation employs low tidal volumes to reduce volutrauma and positive end expiratory pressure (PEEP) to maintain patency (Acute Respiratory Distress Syndrome Network, 2000).

The organ-scale mechanical effects of lung injury are driven by alterations in the mechanical function of the alveoli and alveolar ducts that comprise the lung parenchyma (Knudsen et al., 2023). These changes in the structure-function relationship potentiate VILI. Alveolar derecruitment (atelectasis), caused by edema and elevated surface tension, reduces the effective size of the lungs [‘baby lung’, (Gattinoni and Pesenti, 2005)] and causes stress concentrations (Mead et al., 1970) leading to increased tissue strains and volutrauma. Surfactant dysfunction also causes alveolar instability (Knudsen and Ochs, 2018) that drives atelectrauma through the repeated alveolar collapse and reopening and potentially by the folding and unfolding of septal pleats (Ruhl et al., 2019). At the broadest level, this means that the injured lung is more prone to VILI than the healthy lung (Mellenthin et al., 2019).

A more nuanced perspective is that lung-protective ventilation settings should be guided by the mechanical function of the parenchyma to reduce the damaging microscale stresses and strains imposed by the ventilator (Nieman et al., 2017). However, acute lung injury (ALI) and ARDS are broad categories, and different injury etiologies have profoundly different effects on the lungs. Therefore, in order to develop new approaches to protect the injured lung, we must understand the effects of different injury modes on the structure and function of the parenchyma.

In the current study, we consider uninjured controls and three mouse lung injury models. Intratracheal (IT) endotoxin (lipopolysaccharide, LPS) instigates an inflammation-dominated injury in a sterile model of sepsis, IT hydrochloric acid (HCL) induces a direct injury to model aspiration of gastric contents or inhalation injury, and an injurious lavage (LAV) models near-drowning. A 2^nd^ ‘hit’ of VILI is applied in all three injury groups to recapitulate the clinical course of injury followed by mechanical ventilation. Lung mechanical function is invasively measured over a broad range of inflation pressures and volume histories with a focus on recruitment and derecruitment dynamics. Parenchymal structure is measured with design-based stereology at three physiologic air-inflation pressures, gene expression of pro-inflammatory markers is measured via quantitative real-time polymerase chain reaction (RT-qPCR) of lung homogenate, and bronchoalveolar lavage protein content provides an estimate of alveolocapillary leak.

## 2 Methods and materials

### 2.1 Animal procedures

Seven-to ten-week-old female C57/BL6 mice (Jackson Laboratories) weighing 16–25.8 g (18.9 ± 1.61 g) were studied under the University of Colorado Denver Institutional Animal Care and Use Committee (IACUC) approved protocol #00230. Injury was induced by intratracheal (IT) instillation of 50 µg lipopolysaccharide (LPS group) from *Escherichia coli* O55:B5 (L4524, Millipore Sigma) or 75 uL of 0.1 M hydrochloric acid (HCl group) 2 days prior to ventilation. The lavage group (LAV) received IT instillation and suction of 0.15 mL saline (LAV) just before ventilation and the control (CTL) group was not injured.

### 2.2 Mechanical ventilation

Prior to ventilation, the mice were anesthetized with an intraperitoneal (IP) injection of 100 mg/kg ketamine and 8 mg/kg xylazine and 2.5 mg/kg acepromazine, tracheostomized with a blunted thin-wall 18-gauge metal cannula and ventilated using a FlexiVent small animal ventilator (SCIREQ, Montreal, QC, Canada). Respiratory drive was suppressed *via* 0.8 mg/kg pancuronium bromide administered at the onset of mechanical ventilation. Alternating doses of 50 mg/kg ketamine and 50 mg/kg ketamine with 8 mg/kg xylazine were administered IP at 30 min intervals.

All injury groups (LPS, LAV, HCl) were subjected to 25 min of ventilation at a plateau pressure of 37.5 cmH_2_O, a positive end expiratory pressure (PEEP) = 0 cmH_2_O, a respiratory rate (RR) = 60 breaths/min, and inspiratory to expiratory ratio (I:E) = 1:1.5 to induce a 2^nd^ hit of ventilator-induced lung injury (VILI). The control group received 6 min of stabilizing baseline ventilation with a tidal volume (Vt) = 10 mL/kg, RR = 150 breaths/min, PEEP = 3 cmH_2_O, and I:E = 1:1.5. During that time recruitment maneuvers (RMs), comprised of a 3 s pressure ramp to 30 cmH_2_O and a 3 s breath hold, were performed at 2-min intervals.

Following stabilization, lung function was assessed in a ‘measurement block’ where an RM was performed and then a quasi-static pressure volume loop (PV) was recorded. The PV loop data was used to calculate the delivered volume from 0 to 30 cmH_2_O (inspiratory capacity, IC), the slope at 5 cmH_2_O on the expiratory limb (quasi-static compliance, Cst), and hysteresis area (A). Ventilation was changed to Vt = 6 mL/kg with I:E = 1:1.5 and the following sequence was conducted at both PEEP = 1.5 cmH_2_O and then 4.5 cmH_2_O. A RM was applied, and then multi-frequency forced oscillations (FOT) were applied 6 times at 20 s intervals to calculate mechanical impedance which was then fit to the constant phase model (Hantos et al., 1992) to determine respiratory system elastance (H), tissue damping (G), and Newtonian resistance (Rn). Hysteresivity (η) = G/H was calculated to describe the balance between tissue damping and elastance, and elevated values of η may indicate increased ventilation heterogeneity (Bates, 2009). Twenty seconds of ventilation were then recorded at four different settings: 1) Vt = 8 mL/kg, RR = 190 breaths/min, I:E = 1:1.5, 2) Vt = 8 mL/kg, RR = 187.5 breaths/min, I:E = 1:1, and a 0.08 s inspiratory hold, 3) Vt = 10 mL/kg, RR = 150 breaths/min, I:E = 1:1, and a 0.1 s inspiratory hold, and 4) driving pressure (P_D_) = 10 cmH_2_O, RR = 150 breaths/min, PEEP = 0, I:E = 1:1.5. Note that these four ventilation patterns are not used in the current analysis.

After the measurement block, a ‘PEEP ladder’ was recorded with PEEP starting at 0 cmH_2_O and incrementing in steps of 3 cmH_2_O to a maximum value of 15 cmH_2_O, before being returned to 0 in steps of 3 cmH_2_O. One minute of ventilation was applied at each PEEP and FOT measurements were recorded at the start, middle, and end of each 1-min period. We refer to the increasing PEEP phase as the ‘ascending’ ladder, and the decreasing PEEP phase as the ‘descending’ ladder. Three PEEP ladder ventilation patterns were investigated in separate cohorts of mice: 1) the VT6 subgroup (Vt = 6 mL/kg, RR = 250 breaths/min, I:E = 1:1.5), 2) the VT6RMs subgroup where an RM was applied at each PEEP change and then VT6 was used, or 3) the VT10 subgroup (Vt = 10 mL/kg, RR = 150 breaths/min, I:E = 1:1).

Finally, a second repetition of the measurement block was applied to assess changes during ventilation, and then the lungs were harvested for morphometric or biomarker analysis. Total ventilation time was ≈35 min for mice that did not receive VILI and ≈1 h for mice that did receive VILI.

Each mouse was instrumented with a custom respiratory flowmeter based on Jawde *et al* (Jawde et al., 2018). Here, the flow-induced pressure differential down a channel with a resistance between 0.25 and 0.3 cmH_2_O/ml/s was used to determine the flow rate at the tracheal opening. Those data are not included in this report.

### 2.3 Morphometry (stereology)

#### 2.3.1 Tissue preparation

Lung structure was assessed using design-based stereology (Mühlfeld et al., 2013) which is the American Thoracic Society gold standard for structural quantification (Hsia et al., 2010). The lungs were fixed through the vasculature while the air inflation pressure was held at a prescribed level (described below) to maintain surface tension effects and allow direct comparison to the lung function data (Gil et al., 1979; Bachofen et al., 1982). Following ventilation, a bilateral thoracotomy was performed, and the pulmonary circulation was flushed with ≈5 ml of 100 U/ml heparinized saline with 3% 100 kDa dextran at a pressure of 35 cmH_2_O. Three RMs were then applied and then airway pressure was held at 30 cmH_2_O for 3 seconds before ramping down to either 2, 5, or 10 cmH_2_O and ligating the trachea. The lungs were then perfused with 5 mL of 4% paraformaldehyde, 1% glutaraldehyde, and 3% dextran in 0.15 M HEPES buffer, carefully excised, and immersion fixed at 4°C for at least 24 h. Somewhat unexpectedly, lungs from the HCL group showed volume loss during the immersion fixation. For that cohort, a continuous pressure of 2, 5, or 10 cmH_2_O was applied during the immersion fixation using a low-flow pump and an oil column to set the prescribed pressure. At no point were the lungs allowed to deviate from that set pressure.

Extrapulmonary tissue was trimmed away, and the total lung volume was measured using water displacement. Lungs were embedded in 3% agar and 2 mm slabs were cut using a thin, sharp blade. Even or odd numbered slices were selected by coin flip, washed in 0.15 M HEPES, and vacuum degassed. The lung slices were then washed 4 × 5 min in 0.1 M cacodylate buffer, post-fixed in 1% osmium tetroxide for 2 h at 4C, washed 4 × 5 min in 0.1 M cacodylate and once in water, and then incubated overnight in 4% uranyl acetate.

The uranyl acetate-treated tissue was rinsed 4 × 5 min in distilled water and then dehydrated in a Lynx II tissue processor (Electron Microscopy Sciences) for 2 × 10 min in 70% acetone, 2 × 10 min in 80% acetone, 2 × 10 min in 90% acetone, and finally 3 × 10 min in 100% acetone. The tissue was infiltrated for 2 h with 50% acetone and 50% Technovit 7,100 and then overnight with 100% Technovit 7,100 at 4°C. Finally, the tissue was embedded in Technovit 7100 as described by the manufacturer, mounted to posts with Technovit 3040, sectioned at 1.5 µm with a tungsten carbide blade, transferred onto glass slides, and stained with a toluidine blue.

#### 2.3.2 Morphometric analysis (stereology)

Whole-slide images were scanned with a ×20 objective on an Olympus BX53 with a Prior motorized stage and z-drive using the Olympus CellSens software. The whole-slide images were imported into the NewCAST stereology software (Visiopharm) and systematic uniform random sampling was conducted to gather 100–200 representative fields of few at 5x, 20x, and 40x digital magnification. A cascade sampling design and point counts were used to estimate volume fractions. Starting at 5x, the total lung volume was subdivided into parenchymal (Vv(Par)) and non-parenchymal fractions. The parenchymal volume (V(Par)) was then calculated by multiplying Vv(Par) by the lung volume measured with water displacement. At 20x, the parenchyma was divided into the alveolar, the alveolar duct airspace, and non-air volume fractions. Those quantities were multiplied by V(Par) to determine the volume non-air material in the parenchyma (V(ParNonAir)) as well as the alveolar airspace volume (V(AlvAir)) and the alveolar duct airspace volume (V (DuctAir)) which are summed together to yield the volume of air in the parenchyma (V(ParAir)). At 40x, V(ParNonAir) was divided into volumes of airspace edema (V (Edema)), aerated inter-alveolar septa (V(Septa, Open)), and collapsed inter-alveolar septa (V(Septa, Collapsed). Collapsed inter-alveolar septa were identified by multiple layers of capillaries, indicating that two or more septa (each with a single capillary layer), were ‘piled up’ due to alveolar collapse or septal folding as depicted in the inset of Figure 7B. The chord length across parenchymal airspaces, including both the alveolar and ductal compartments, was manually measured 600–700 times per animal and used to generate kernel density functions (airspace size distributions) and calculate the mean linear intercept length (MLI). Line probes were also used to estimate the surface area density (area per volume) in the parenchyma which was multiplied by V(Par) to determine the gas-exchanging surface area (S(Septa)). More detail on the stereological approach is available in our previous publications, e.g., (Knudsen et al., 2018; Smith et al., 2020; Rizzo et al., 2021).

### 2.4 Gene expression

Whole lungs were harvested, homogenized, and Qiazol phase separation RNA extraction was performed. Briefly, whole lungs were homogenized in 2 mL Qiazol reagent using gentleMACs tissue dissociator and then centrifuged at 300 x g for 5 min. The supernatant was transferred to the tubes containing 0.4 mL chloroform and incubated for 2–3 min. After, solutions were centrifuged at 12,000 x g for 15 min at 4°C, then the aqueous phase containing RNA was transferred to a new tube and stored at −80°C until needed for RT-qPCR gene expression analysis. One step RT-qPCR was performed using Quantinova RT-qPCR Qiagen kit (#208354) according to the manufacturer’s instructions. Briefly, RNA was quantified using nano-drop and normalized to 125 ng/μL with a final concentration of 250 ng per reaction. ThermoFisher Taq primers for TNF-α, IL-6, CXCL-1, NF-κβ, and GAPDH were used. Gene expression was calculated using Δ-ΔCt log_2_ fold change.

### 2.5 Airspace protein content

BALF was collected by instilling and suctioning of 0.8 mL 1xPBS with 100 µM EDTA and protease inhibitor through the tracheal cannula twice, for a total of 1.6 mL. BALF was centrifuged at 400 x g for 7 min; supernatant was transferred to a new tube and stored at −80°C. Total protein concentrations of BALF were measured using the Pierce BCA Protein Assay kit (CAT #23225) according to the manufacturer’s instructions.

### 2.6 Statistical analysis

Data was curated in MATLAB version 2022a and the statistical analysis was performed in RStudio version 2021.09.1. The lung mechanics and stereological data was analyzed with a linear mixed effects model using the *lmer* function in the *lme4* package version 1.1–27.1. Fixed effects for the PEEP ladder mechanics were the experimental group, the measurement PEEP, the ascending or descending limb of the ladder, and (if relevant) the PEEP ladder ventilation pattern. Fixed effects for mechanics measurements before and after the PEEP ladder were the experimental group and the timing of the measurement (before or after the ladder). Fixed effects for the stereological data were the experimental group and the fixation airway pressure. In all cases, the intercept for each animal was included as a random effect. The mean intercept lengths were analyzed with a linear model (*lm* in the R *stats* package version 4.1.2). Once the linear models were fit, the marginal means were computed with *emmeans* (version 1.7.0) and the *contrast* function, which is *emmeans* package, was used to for pairwise significant differences between groups. The false discovery rate was used to account for multiple comparisons.

A multinomial logistic ridge regression was performed to identify the relative importance of the different lung function measurements in predicting experimental group membership using the *glmnet* package in MATLAB (Qian et al., 2013). In this analysis, the regularization parameter (λ) is iteratively adjusted and we report the normalized regression coefficients (β) for λ that yields the minimum error between the known experimental groups and model predictions in 20-fold cross validation. Separate training and testing datasets were not used due to the relatively small sample size.

Airspace intercept lengths were analyzed by computing the kernel probability density estimates using the *ksdensity* function in MATLAB. Bootstrapped confidence intervals were estimated by computing 1,000 probability density estimates of the dataset resampled with replacement and identifying the 2.5% and 97.5% quantiles with the *quantile* function in MATLAB.

## 3 Results

### 3.1 Lung mechanical function

**FIGURE 1:**
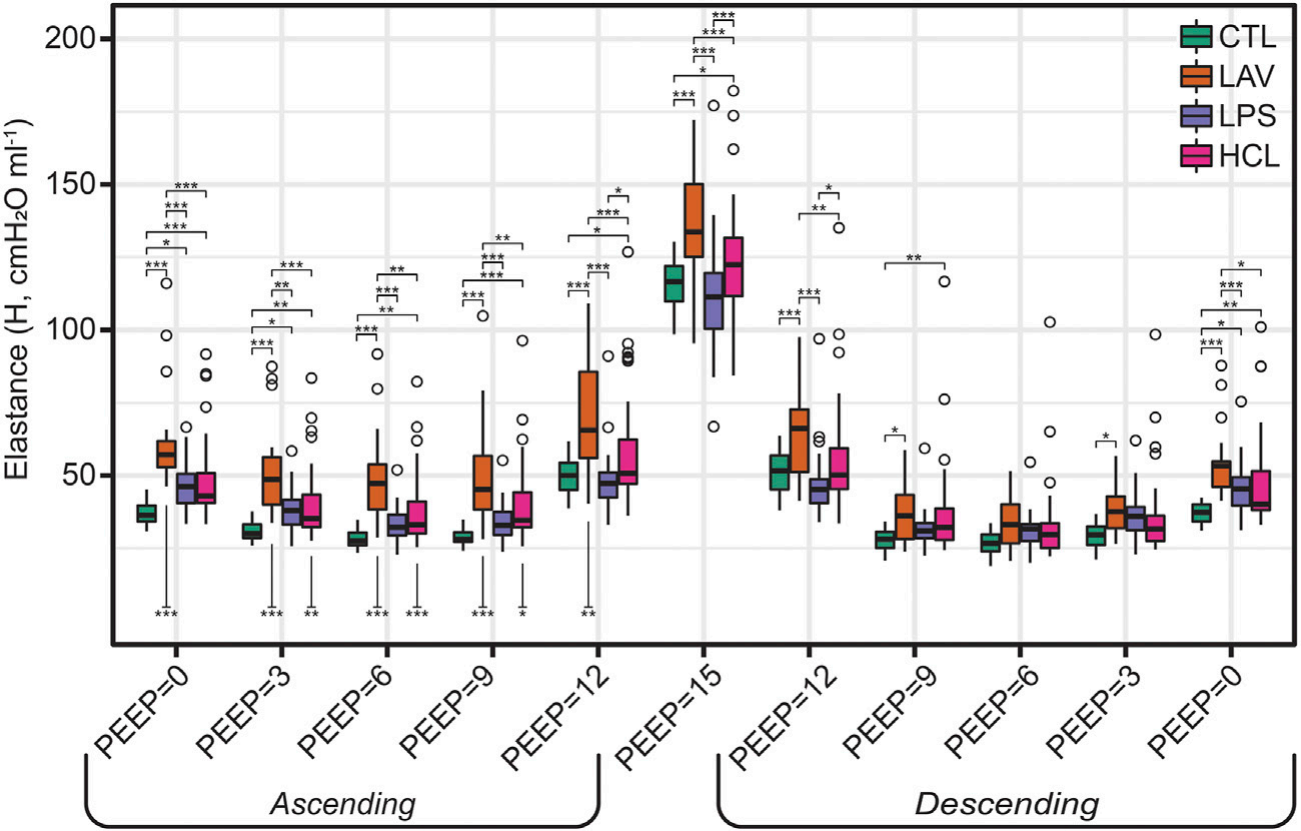
Pulmonary system elastance (H) measured during the PEEP ladders with PEEP incrementing from 0 to 15 cmH_2_O (Ascending) and then decreasing from 15 to 0 cmH_2_O (Descending). Symbols above the bars indicate significant inter-group difference at that PEEP and indicate injury-induced stiffening. Symbols below bars indicate significant intra-group differences between measurements recorded on the Ascending and Descending limbs of the PEEP ladder and are indicative of recruitment.

**FIGURE 2:**
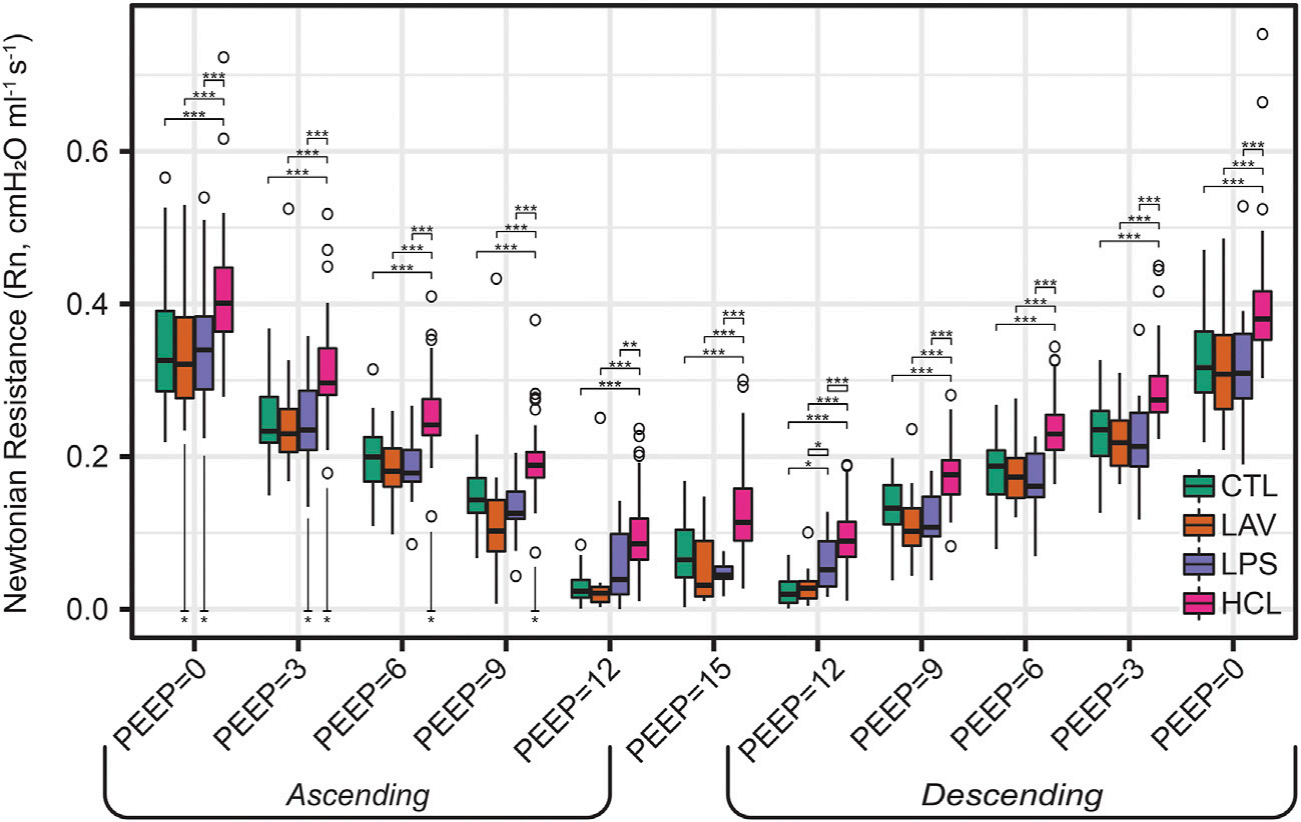
Newtonian resistance (Rn) measured during the PEEP ladders with PEEP incrementing from 0 to 15 cmH_2_O (Ascending) and then decreasing from 15 to 0 cmH_2_O (Descending). Symbols above the bars indicate significant inter-group difference at that PEEP; symbols below bars indicate significant intra-group differences between measurements recorded on the Ascending and Descending limbs of the PEEP ladder.

**FIGURE 3:**
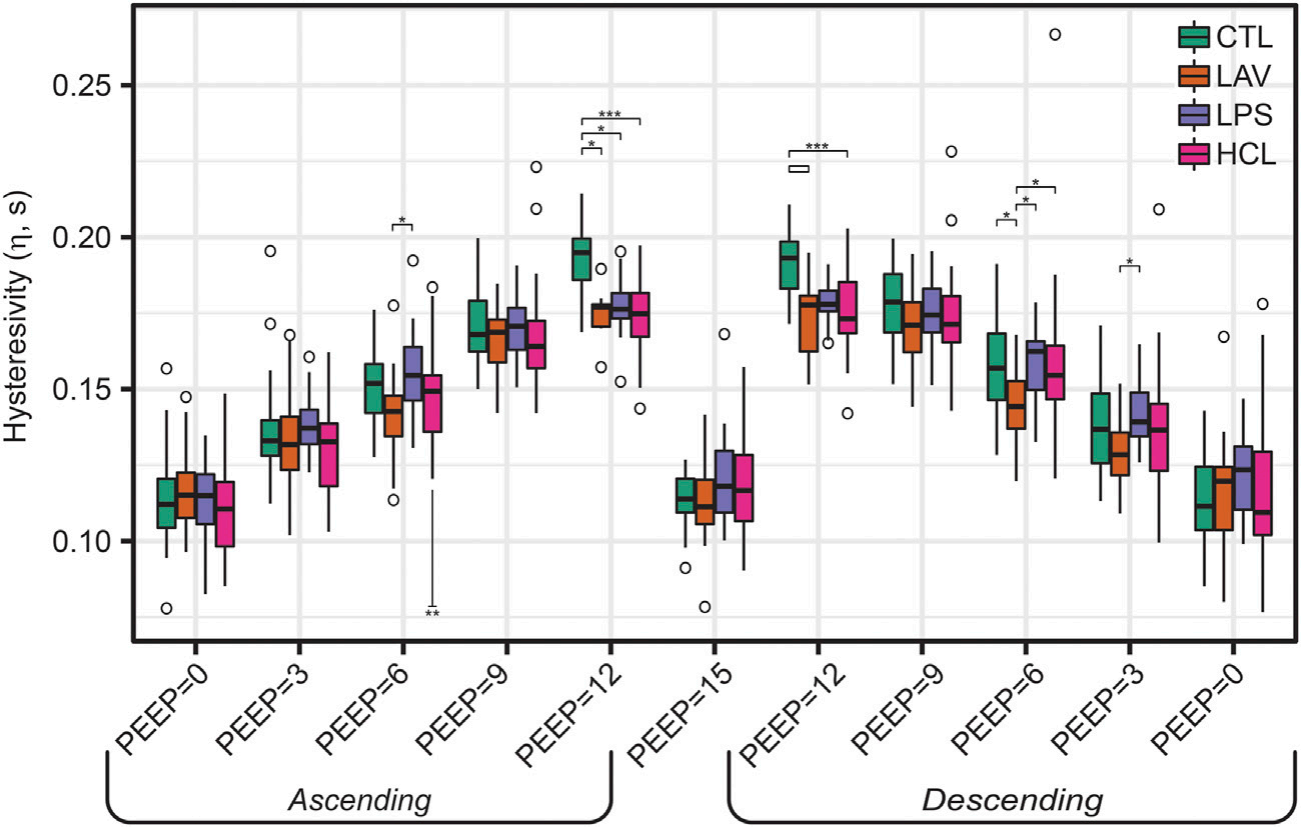
Hysteresivity (η = G/H) measured during the PEEP ladders with PEEP incrementing from 0 to 15 cmH_2_O (Ascending) and then decreasing from 15 to 0 cmH_2_O (Descending). Data for G is shown in Supplementary Figure S1. Symbols above the bars indicate significant inter-group difference at that PEEP; symbols below bars indicate significant intra-group differences between measurements recorded on the Ascending and Descending limbs of the PEEP ladder.

The centerpiece of the lung mechanics measurement protocol is a PEEP ladder where the PEEP was incrementally increased from 0 to 15 and back to 0 cmH_2_O in steps of 3 cmH_2_O. At each PEEP, forced oscillation measurements were recorded to compute pulmonary system elastance (H, Figure 1), Newtonian resistance (Rn, Figure 2), and tissue damping (G, Supplementary Figure S3) from which hysteresivity (η) = G/H (Figure 3) is calculated. We will briefly introduce those PEEP ladder data to clarify the measurement sequence and highlight important trends that are shared across the groups. Then we will use a ridge regression analysis to highlight important inter-group differentiators for further discussion.

**FIGURE 4:**
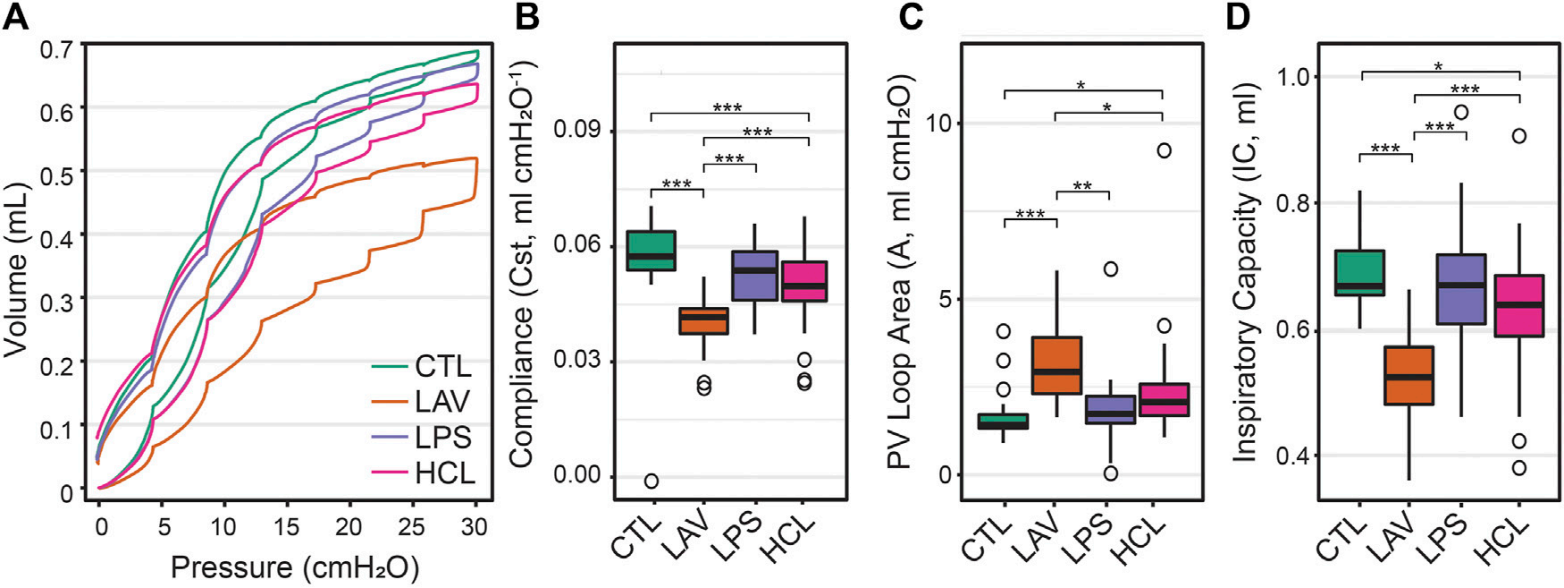
Quasi-static pressure-volume loops recorded before the PEEP ladders **(A)**. The quasi-static compliance (Cst, panel **(B)**, PV loop hysteresis area (A, panel **(C)**, and the volume delivered during the PV loop (IC, panel **(D)** calculated from the raw pressure-volume loops. PV loop data recorded after the PEEP ladders is provided in Supplementary Figures S2–S4.

Figure 1 shows elastance as PEEP is increased (Ascending) and decreased (Descending) during the PEEP ladder. Note that in Figures 1–3, the mice from the VT6, VT6RMs, and VT10 ventilation subgroups are pooled, and those groups are broken out for further analysis in Figure 6. Starting at PEEP = 0 and increasing PEEP, elastance tends to decrease until PEEP≈6 in all groups. Then, due to the strain-stiffening properties of the pulmonary system (shown in the PV loops in Figure 4A), elastance begins to increase up to PEEP = 15 where the highest H was recorded for all groups. On the descending limb, a similar trend is observed with elastance decreasing until PEEP ≈ 6, and then increasing again as PEEP is further decreased to zero. Note that in LAV and HCL the elastance is significantly higher at some PEEPs on Ascending than Descending, suggesting recruitment by ventilation at high PEEP. Those Ascending-Descending differences are indicated by asterisks below the relevant bars on the Ascending side of the plot.

The trends in Rn (Figure 2) are markedly different. Here, we observe an inverse correlation between Newtonian resistance and PEEP, with Rn decreasing from PEEP = 0 to PEEP = 15 and then increasing as PEEP is reduced back to zero on the Descending limb of the ladder. As with H, changes in Rn at a given PEEP from the Ascending to Descending limb of the ladder are demarcated by asterisks below the relevant bars on the Ascending side of the plot. Those high-pressure-induced changes are found in all injury groups but are most prevalent in HCL.

Hysteresivity (Figure 3) increases linearly with PEEP on the ascending limb, indicating that tissue damping (G) is increasing more rapidly than H. This trend holds until PEEP = 15 cmH_2_O, where there is a marked decrease in η. Given that H is at a maximum here, this indicates that G increased less rapidly than H when compared to PEEP = 12 (see Supplementary Figure S1). Starting at PEEP = 12 on the descending ladder there is a steady decrease in η with decreasing PEEP, mirroring the changes on the Ascending ladder.

Quasi-static pressure volume loops, recorded prior to the PEEP ladder, are shown in Figure 4 and exhibit the characteristic high-volume stiffening ‘beak’ at pressures ≳ 15 cmH_2_O (Figure 4A). The LAV group has the most distinctive PV loop showing decreased quasi-static compliance (Cst, Figure 4B), hysteresis area (A, Figure 4C), and lowest delivered volume (IC, Figure 4D). These trends are present, but reduced, in HCL and further attenuated in LPS. PV loop parameters measured after the PEEP ladders are shown in Supplementary Figures S2–S4.

Given the complexity of the lung mechanics dataset which has more than 60 different permutations of measurement parameters and PEEPs per group, we performed a multinomial ridge regression to identify important predictors of experimental group membership. The confusion matrix showing the regression model performance is provided in Supplementary Figure S5. We will use this analysis to steer discussion of how the details of mechanical function differ between the experimental groups.

**FIGURE 5:**
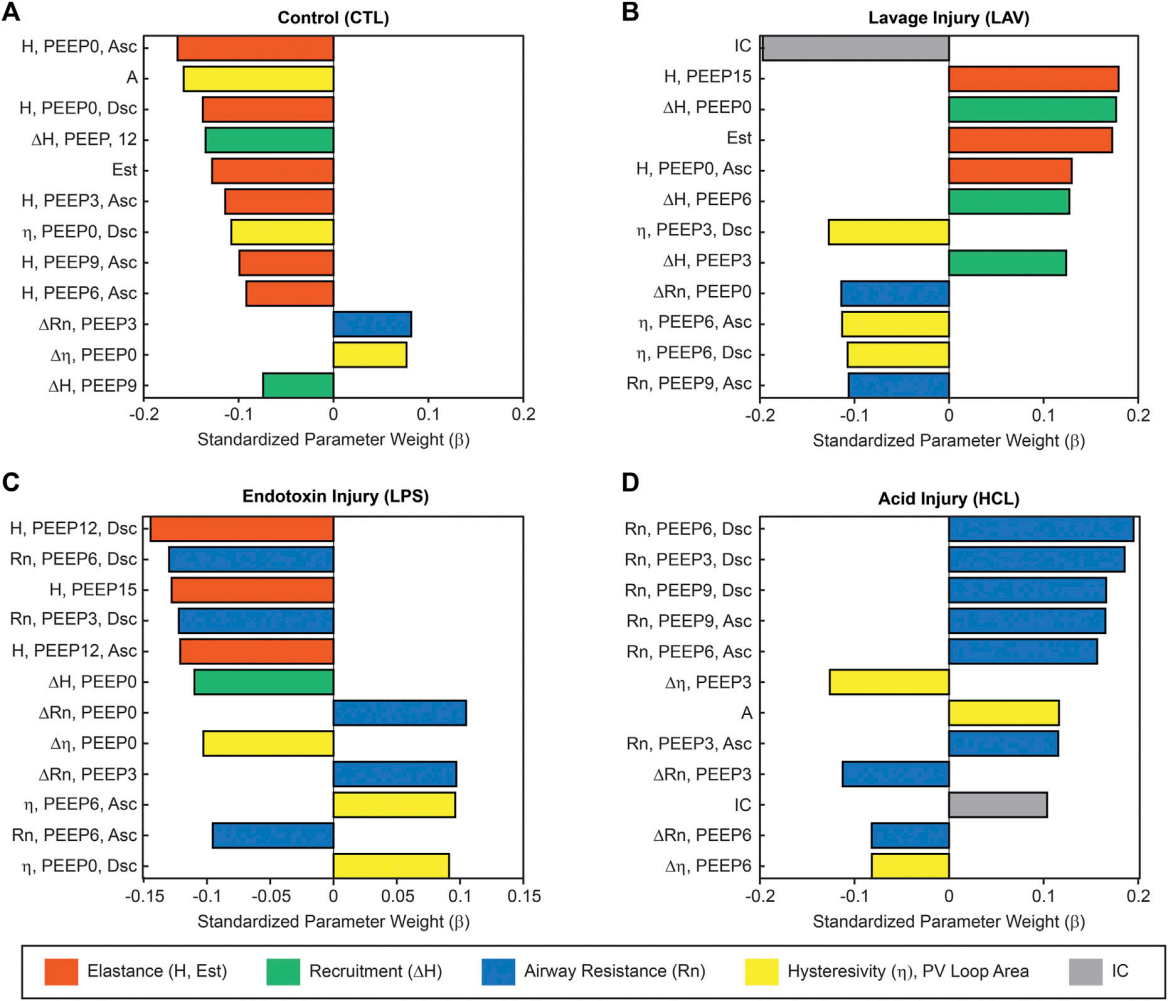
Ridge regression of the complete lung mechanics dataset showing the normalized regression parameters rank-ordered by magnitude. Large positive values indicate that high values for that parameter are an important identifier for that experimental group. Large negative values indicate an inverse correlation. This analysis reveals the relative importance of each parameter in classifying experimental group membership for CTL **(A)**, LAV **(B)**, LPS **(C)**, and HCL **(D)**. Colors indicate parameters related to stiffness (orange), recruitment (ΔH, green, the change in elastance from the Ascending to Descending ladders), Newtonian resistance (blue), hysteresivity and PV loop area (yellow), and PV loop delivered volume (grey). Parameters from the PEEP ladders are described by the parameter name (H, Rn, or η), the measurement PEEP, and if the measurement was recorded on the Ascending (Asc) or Descending (Dsc) limb of the PEEP ladder.

Figure 5 shows the normalized regression coefficients (β) rank-ordered by magnitude. Here, high positive values of β indicate that high values of a particular measurement are an important differentiator for that group, e.g., high PEEP = 15 cmH_2_O elastance is characteristic of LAV. Likewise, high-magnitude negative β values indicate that an experimental group is characterized by low values for that measurement, as in the case in the PV Loop delivered volumes (IC) for LAV. The model parameters are ordered by magnitude so that each panel shows the 12 most important parameters for identifying that group. The full set of coefficient values for each group are provided in Supplementary Figures S6–S8.

The ridge regression coefficients show that, in comparison to the other groups, the Controls (Figure 5A) are characterized by distensible, stable lungs. Elastance measured with the FOT (H) is low over the range of measurement PEEPs; Figure 1 shows CTL H is lower than the injury groups at all PEEPs, particularly on the Ascending limb of the PEEP ladder. PV loop quasi-static elastance (Est = 1/Cst) is also low (Figure 4C). There is little recruitability (ΔH) which is the change in elastance at a given PEEP from the Ascending to Descending limb of the ladder.

In contrast, the LAV group (Figure 5B) is classified by small, stiff, and unstable lungs. PV loop volumes are low (IC, Figure 4C) and H is significantly higher than all other groups during the ascending ladder from PEEP = 0 cmH_2_O (H, PEEP0, Asc) all the way up to PEEP = 15 cmH_2_O (H, PEEP15) as detailed in Figure 1. Recruitability (ΔH) is identified as an important classifier at PEEP = 0, 3, and 6 cmH_2_O, indicating that after ventilation at higher PEEPs the elastance is substantially reduced at those measurement PEEPs on the descending limb of the PEEP ladder. Significant changes between Ascending and Descending H are indicated in Figure 1 by asterisks bellows the bars. Low hysteresivity (η = G/H) is also an important classifier for LAV, indicating that H increased more than tissue damping (G). Figure 3 shows that low hysteresivity is most prominent (and significant) in LAV on the descending limb of the ladder.

For LPS (Figure 5C), the regression analysis indicates that H is relatively low at high PEEPs and only modestly elevated for PEEP ≤3 cmH_2_O. Figure 1 shows that H is higher for LPS than CTL at low PEEPs and then the two groups are similar for PEEPs ≥6 cmH_2_O. This is in contrast to the LAV and HCL injuries where elevated elastance on the Ascending limb of the PEEP ladder persists to PEEP = 15. On the descending limb, the differences in elastance are muted until low PEEPs. Hysteresivity in LPS is similar to CTL, and significantly higher than in LAV and HCL at some PEEPs (Figure 3). Low Newtonian resistance (Rn) is a strong differentiator of LPS from HCL (Figure 2), and Rn is similar between LPS, CTL, and LAV. Interestingly, the LPS group is characterized by a decrease in Rn on the descending limb of the PEEP ladder (ΔRn) as shown for PEEP = 0 and PEEP = 3 in Figure 2, indicating a high-pressure-induced airways enlargement.

The HCL group is most strongly differentiated by increased Rn that suggests damage to the conducting airways by the intratracheal acid instillation. This is shown in Figure 2 where HCL has significantly elevated Rn compared to all other groups at all PEEPs on both the ascending and descending limbs of the ladder. It is important to note that H does not appear as a top classifier of HCL in the ridge regression, but Figure 1 clearly shows that H is elevated for HCL when compared to CTL, particularly on the ascending limb of the ladder. It is not considered to be one of the most important classifiers because H falls between CTL, LAV, and LPS while Rn is significantly higher for HCL at all PEEPs. Finally, the PV loop area and IC are important classifiers of HCL when considered in conjunction with the other regression model parameters. However, it should be noted that these parameters are higher in LAV than HCL (Figure 4).

**FIGURE 6:**
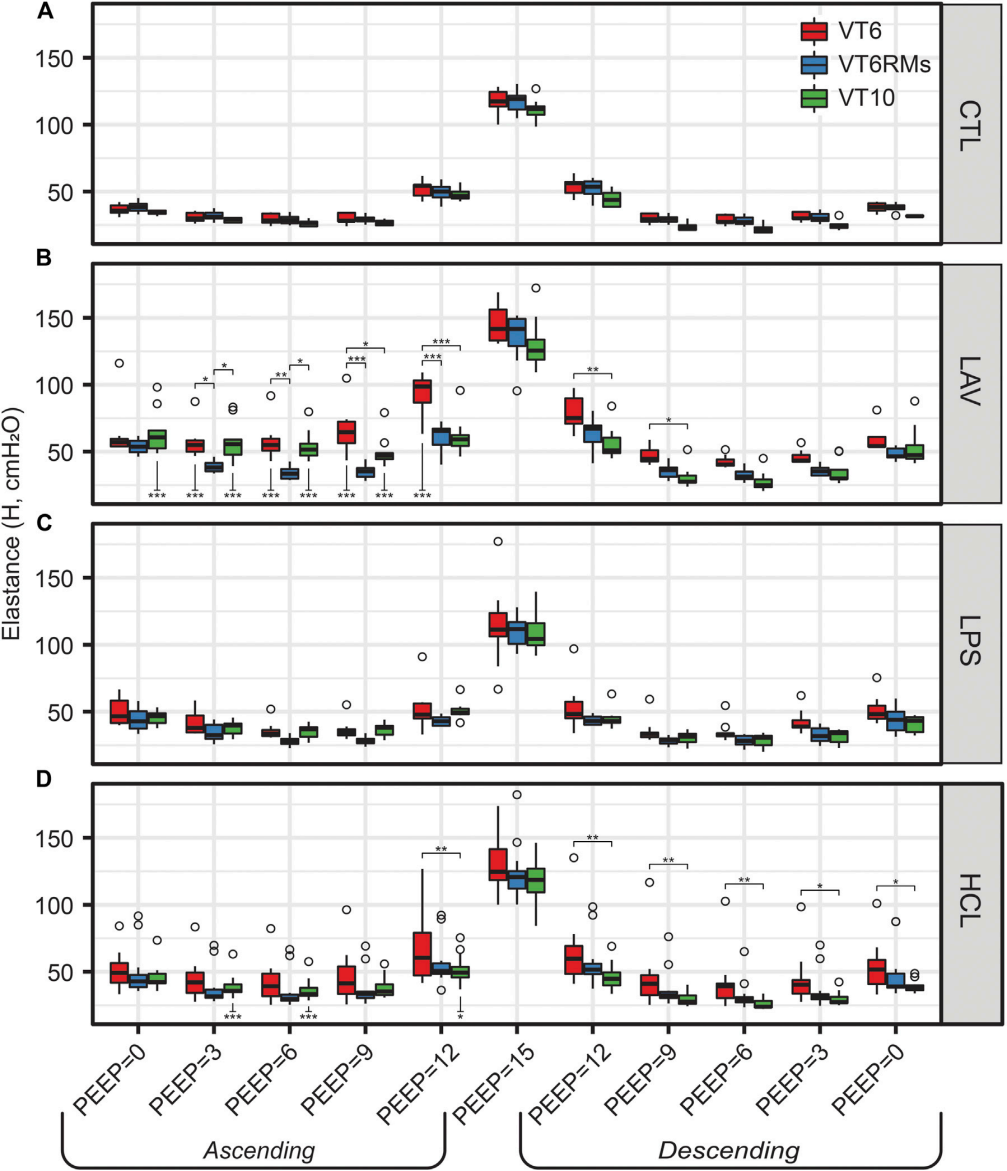
Elastance (H) for the four experimental groups [CTL **(A)**, LAV **(B)**, LPS **(C)**, HCL **(D)**] and three ventilation patterns applied during the PEEP ladders (bar colors). Symbols above the bars indicate significant difference between ventilation patterns at that group and PEEP; symbols below bars indicate significant differences between measurements for the same group and ventilation pattern at the same PEEP recorded on the Ascending and Descending limbs of the PEEP ladder.

Figures 1–3 show the three ventilation groups (VT6, VT6RMs, and VT10) pooled together for each treatment group. Figure 6 shows H measured during the PEEP ladders with each treatment in a separate panel and different ventilation types indicated by bar color. Controls and LPS are notable because there are no effect of ventilation pattern on H. In contrast, higher tidal volume ventilation (VT10 vs. VT6) yielded lower H on the descending ladder in HCL, although this difference is modest.

The behavior in LAV is most interesting. Starting at PEEP = 0 on the Ascending ladder, all ventilation types have the same H. With increasing PEEP, H first decreases in the VT6RMs group at PEEP = 3, which we attribute to recruitment from the brief excursion to 30 cmH_2_O (the RM) and sufficient PEEP to maintain patency. Next, we see the VT10 group begin to recruit at PEEP = 9 and achieve the same recruitment at the VT6RMs group at PEEP12, which we attribute to the higher airway pressures at VT10 compared to VT6. At PEEP15, H is identical for all groups because airway pressures in VT6 are sufficient to achieve recruitment. On the descending ladder, VT6 has higher H than VT6RMs and VT10 at all PEEPs.

### 3.2 Lung structure

**FIGURE 7:**
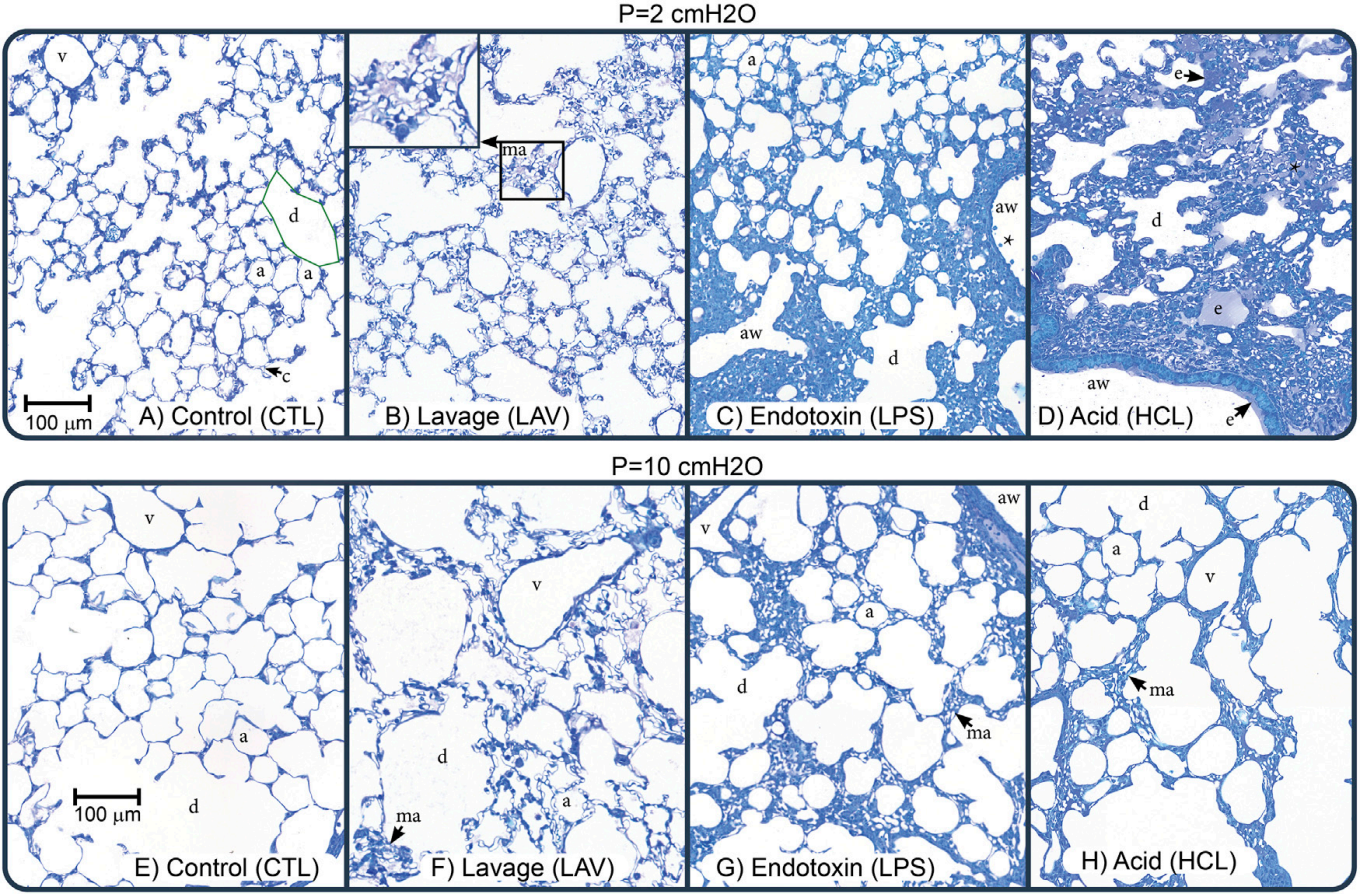
Representative micrographs showing perfusion-fixed lung tissue that was air-inflated at *p* = 2 cmH_2_O (1^st^ row) or *p* = 10 cmH_2_O (2^nd^ row). The control (CTL), lavage (LAV), endotoxin (LPS), and acid injury (HCL) groups are shown in the 1^st^ through 4^th^ columns. Annotations highlight alveoli (a), alveolar ducts (d), traced with green in panel **(A)**, microatelectases (ma, shown in inset in panel **(B)**, airspace edema (e), septal capillaries (c), conducting airways (aw), large vessels (v), and cell infiltrates (*).

Figure 7 shows representative images for each experimental group (columns) at air inflation pressures *p* = 2 and *p* = 10 cmH_2_O (rows). At *p* = 2, the CTL lungs (Figure 7A) are homogenously inflated with patent alveoli (a) and alveolar ducts (d) that are defined by the free edge of the alveolar septa (green line). The septal capillaries (c) are large, open, and free of cells due to the vascular perfusion fixation. With LAV injury (B), microatelectases (ma) are visible throughout the lungs. These regions of collapse were identified by the ‘stacked’ capillary layers that are indicative of two or more layers of alveolar septa that are ‘piled up’ (inset in panel B) (Rizzo et al., 2021; Wallbank et al., 2023). Endotoxin injury (C) was quite heterogeneous and primarily consisted of large swaths of collapse adjacent to the airways (aw) along with cell infiltrates (*). These injured regions were interspersed with regions of relatively normal appearing alveoli. Acid injury (D) showed more airspace edema (e) which contained cell infiltrates and was found mostly in regions with microatelectases. This field of view also shows a thin layer of edema (e) in the airway (aw). Representative images of whole lobes are provide in Supplementary Figure S10.

Increasing inflation pressure to *p* = 10 cmH_2_O (Figures 7E–H) increased alveolar size and yielded less prominent septal capillaries in uninjured regions which we attribute to increased compressive forces caused by the elevated pressure. This capillary flattening was less prevalent in LAV, which we attribute to increased surface tension forces exerting traction on the septal walls. In the LAV group, microatelectases were less frequent than at *p* = 2 cmH_2_O but still widespread. In both LPS and HCL there was not a marked change in recruitment with increasing pressure and regions or patent alveoli showed pressure-induced enlargement and thinning of the septal capillaries.

**FIGURE 8:**
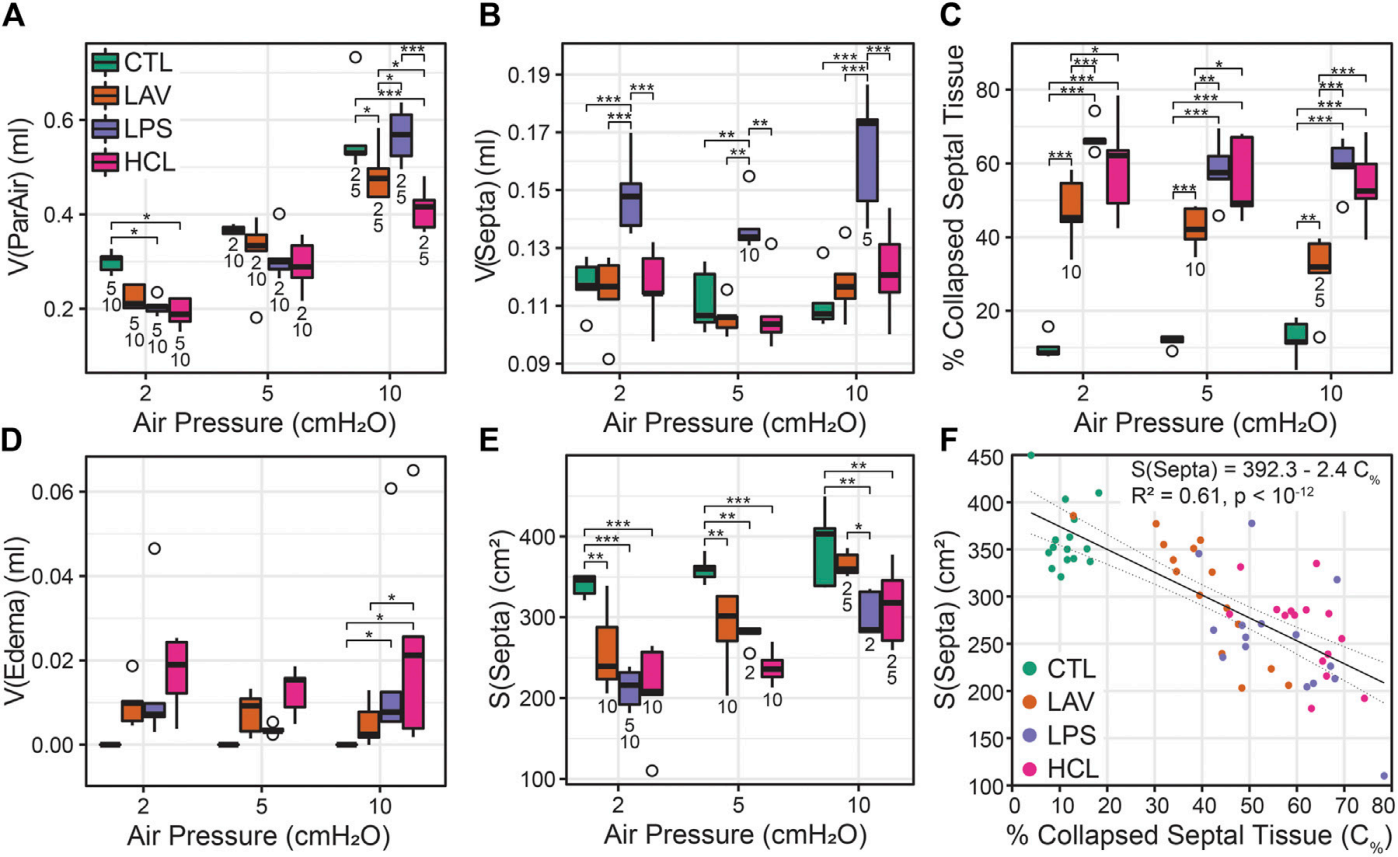
Lung morphometry (stereology) performed at air inflation pressures of *p* = 2, 5, and 10 cmH_2_O (horizontal axes); the four experiment groups are shown in green (CTL), orange (LAV), lavender (LAV), and magenta (HCL). The volume of parenchymal air [V(ParAir), **(A)**] is comprised of gas in the alveoli and alveolar ducts. The volume of septal tissue in the parenchyma [V(Septa), **(B)**] includes both septa from both patent and collapsed alveoli that were identified by multiple layers of alveolar capillary network that were ‘piled up’. The percentage of collapsed septal tissue is shown in **(C)**. The volume of edema in the airspace is shown in **(D)**. The gas-exchanging surface area [S(Septa), **(E)**] is the surface area of septa that was exposed to air and not covered with edema visible at the light microscopy level. The relationship between the % of collapsed septal tissue and S(Septa) **(F)** shows a strong linear correlation. Significant differences at each pressure are indicated with symbols above the bars; significant differences between pressures for each group are indicated with number below the bars (the number indicates the pressure that data is different from).

Injury- and pressure-induced structural changes were quantified with design-based stereology (Figure 8). The volume of parenchymal air, which includes the alveolar and alveolar duct airspaces, increased with pressure in all groups (V(ParAir), Figure 8A) as indicated with numbers below the bars. Marked inter-group differences were not noted until pressure reached 10 cmH_2_O, where CTL and LPS showed significantly higher volumes than LAV and, to a greater degree, HCL. The total volume of alveolar septal tissue (V(Septa), Figure 8B) was significantly elevated in the hyperinflammatory LPS group and was mostly unaffected by pressure. The percentage of collapsed septa tissue (Figure 8C) was calculated as the volume of collapsed septa (Supplementary Figure 11E), identified by multiple layers of septal capillaries ‘stacked up’, divided by the total septal volume. The LPS and HCL injuries had the greatest fraction of septal collapse, which was unchanged by inflation to 30 cmH2O and subsequent deflation to 2, 5, or 10 cmH_2_O, indicating low recruitability. In contrast, the LAV group showed a significant decrease in collapse when comparing *p* = 10 cmH_2_O to *p* = 2 or 5 cmH_2_O.

Airspace edema, quantified in Figure 8D and shown in Figure 7D (e), was significantly elevated in HCL at *p* = 10 cmH_2_O and trended higher at *p* = 2 and 5 cmH_2_O. The septal surface area available for gas exchange (S(Septa), Figure 8E) was significantly reduced for all injury groups at all inflation pressures. For CTL, S(Septa) was not significantly affected by pressures between 2 and 10 cmH_2_O although it did trend upward with pressure. In contrast, the injury groups all demonstrated significant increases in S(Septa) with increasing pressure. Likewise, the parenchymal surface area to volume ratio (Supplementary Figure S11F) was reduced with injury and increased with inflation pressure. This recruitment of gas exchanging surface area may be attributed to the unfolding of septal pleats, the recruitment of whole alveoli, or stretching of the septal walls (Knudsen and Ochs, 2018). The concept that septal surface area is a function of recruitment is bolstered by the strong correlation between the % collapsed septal tissue and S(Septa) (Figure 8F).

**FIGURE 9:**
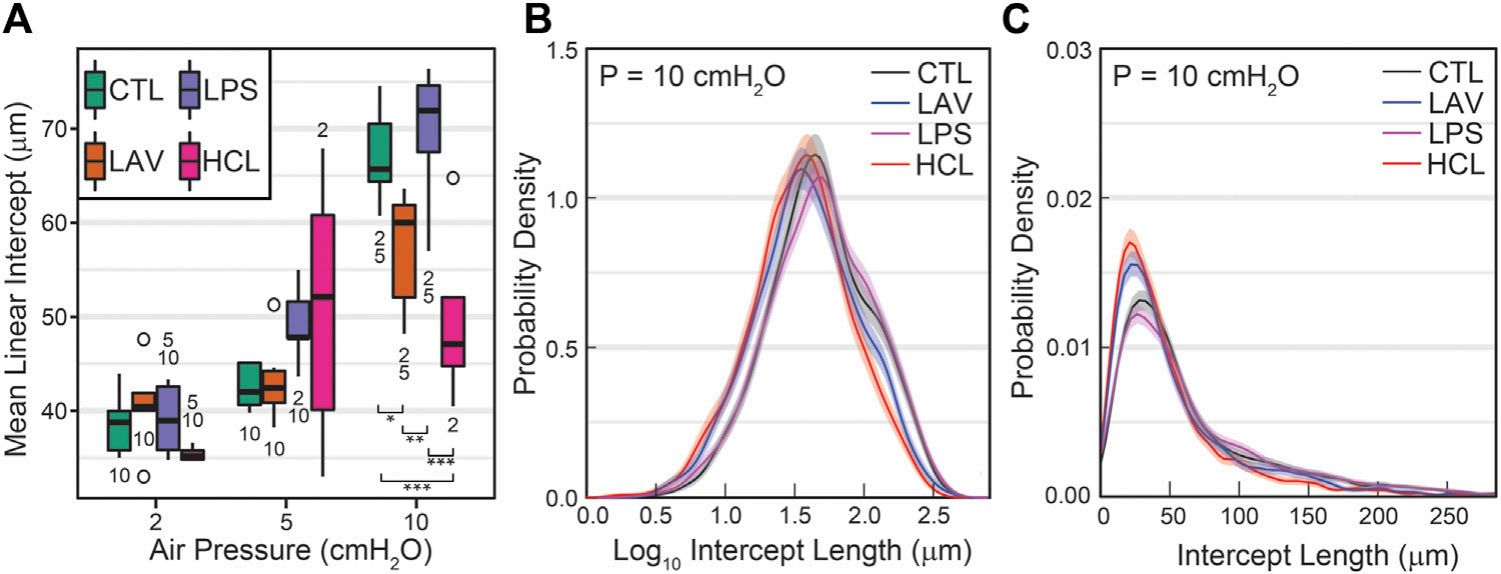
Mean parenchymal airspace intercept length (MLI) at air inflation pressures of 2, 5, and 10 cmH_2_O **(A)**. Significant differences at each pressure are indicated with symbols above the bars; significant differences between pressures for each group are indicated with number below the bars (the number indicates the pressure that data is different from). The average probability density functions for intercept length **(B)** and log_10_ intercept length **(C)** show injury-induced changes in both the small and large airspaces.

The mean linear intercepts (MLIs), a measure of airspace size, are shown in Figure 9A. At *p* = 2 cmH_2_O, there is little difference between the injury groups. Increasing inflation pressure to 10 cmH_2_O yields significant decreases in the mean intercept length for LAV and, to a greater degree, HCL when compared to CTL and LPS. To delve more deeply into these differences we show average probability density functions for intercept lengths (Figure 9B) and log_10_ intercept lengths (Figure 9C) an inflation pressure of *p* = 10 cmH_2_O. In both cases the shaded bands show bootstrapped 95% confidence intervals. The LAV and HCL groups share two common features: 1) and increased probability of small intercept lengths, suggesting that the patent alveoli are reduced in size, and 2) a reduction in larger airspace intercepts ( ≳ 150 µm intercepts), suggesting narrowing of the alveolar ducts. Intercept length distributions for inflation at *p* = 2 and *p* = 5 cmH_2_O are shown in Supplementary Figure 12.

**FIGURE 10:**
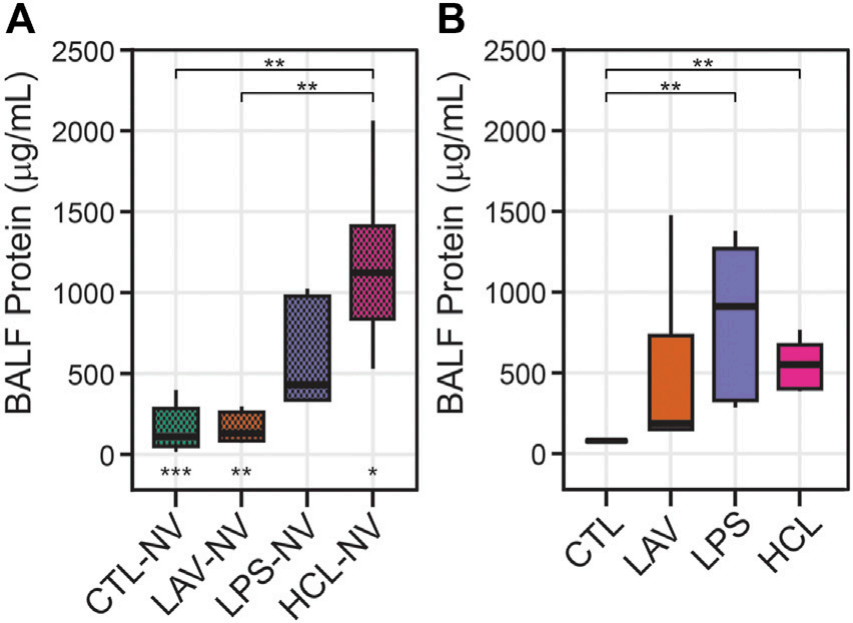
Bronchoalveolar lavage fluid (BALF) total protein content. Panel A shows measurements prior to any ventilation (NV). Panel B shows data immediately prior to the measurement sequence when CTL has been subjected to 6 min stabilizing ventilation and the injury groups have been subjected to 25 min of VILI. Symbols below the bars in A indicate significant ventilation-induced changes (e.g., from **(A, B)**. Symbols above the bars indicate significant inter-group differences within that panel.

These alterations in recruitment (Figure 8C), S(Septa) (Figure 8E), and intercept lengths (Figure 9) may be partially explained by protein in the bronchoalveolar lavage fluid (BALF) causing increased surface tension. Figure 10 shows BALF total protein concentration for each group prior to any ventilation (A), and immediately prior to the measurement sequence (B) after the controls have been stabilized for 6 min (Vt = 10 ml/kg, PEEP = 3 cmH_2_O) and the injury groups have received 25 min of high-pressure ventilation with PEEP = 0 cmH_2_O to induce a degree of VILI. Prior to ventilation, there is no significant difference between CTL and LAV BALF protein (A) which is as expected since there is little tissue injury in LAV prior to ventilation and the alterations in lung function are mainly due to surfactant washout. In contrast, LPS trends upward and HCL is significantly higher than CTL and LAV.

After the initial 6 min stabilization period, the CTL group shows a small but significant decrease in BALF protein (Figure 10B). The effect of 25 min of VILI on the injury groups is much more pronounced, particularly in LAV where there is a significant increase in total protein. LPS also shows a marked increase, although inter-subject variability prevents this from rising to significance. Somewhat surprisingly, BALF total protein was significantly reduced in HCL which may be attributed to inter-subject variability in injury severity.

**FIGURE 11:**
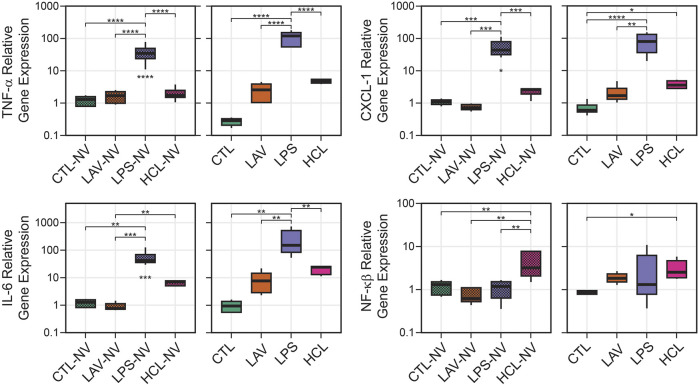
Relative gene expression in lung tissue showing TNF-α, CXCL-1, IL-6, and NF-κB. Data are shown for unventilated animals (NV) and following a brief period of ventilation where CTL received 6 min of stabilizing ventilation and the injury groups received 25 min of injurious ventilation (VILI). Symbols below the bars indicate significant ventilation-induced changes; symbols above the bars indicate significant inter-group differences within that panel.

To understand how different lung injuries affect gene regulation of inflammatory markers, we homogenized whole lungs before any ventilation (NV) and after the initial ventilation period, extracted RNA, and quantified levels of TNF-α, CXCL-1, IL-6, and NF- κβ *via* RT-qPCR (Figure 11). Note that the lavage, used to measure BALF protein, was performed before lung tissue homogenization. As expected, there is little ventilation-induced change in gene expression of inflammatory markers in controls. In the LPS group there are large and significant pre-ventilation increases in expression of TNF-α (Figure 11A), CXCL-1 (Figure 11B), and IL-6 (Figure 11C) which is expected in response to an endotoxin challenge, and the short period of ventilation caused further significant upregulation of those genes. No significant upregulation of NF-κβ (Figure 11D) was observed in LPS prior to, or after, ventilation.

The most prominent feature in HCL was the significant upregulation of NF- κβ expression compared to all other groups before ventilation, and this increase persisted after the short period of VILI although the significance is reduced. The chemokine CXCL-1 was also significantly upregulated in HCL after ventilation, but not before ventilation, indicating that ventilation in this pre-injured group may be promoting increased inflammation and immune cell recruitment. A similar trend with CXCL-1 was observed in LAV but that did not rise to significance. In the LAV group TNF-α, IL-6, and NF- κβ were very similar to CTL prior to ventilation, and trended upward with 25 min of VILI.

## 4 Discussion

Acute lung injury (ALI) and acute respiratory distress syndrome (ARDS) are caused by myriad factors that produce heterogeneous pathophysiologic effects that are incompletely understood. Clinically, ARDS is managed using mechanical ventilation that can lead to ventilator-induced lung injury (VILI) and worse outcomes. In this study, we induced three types of ALI which were then exacerbated by a short (25 min) period of high inspiratory pressure VILI. Injury was quantified with detailed measurements of lung mechanical function, lung structure (stereology), alveolocapillary barrier disruption, and inflammatory gene expression that revealed striking difference between the groups. Briefly, intratracheal endotoxin + VILI induced a hyperinflammatory state that was accompanied by widespread atelectasis but lung stiffness was not severely affected. Intratracheal hydrochloric acid + VILI induced inflammation, alveolocapillary leak, and atelectasis to yield stiff lungs and elevated airways resistance. Lavage injury (surfactant washout) + VILI caused the most dramatic increases in lung stiffness in the absence of inflammation and only a modest degree of alveolar leak.

Intratracheal endotoxin (LPS) is one of the most widely used experimental ALI models and recapitulates the effects of sepsis without live infectious agents, e.g., (Faffe et al., 2000; Chen et al., 2010). In the current study, we intratracheally instilled 50 µg LPS which, as expected, upregulated expression of TNF-α, CXCL-1, IL-6 (Figures 11A–C) and elevated BALF protein (Figure 10). Expression of these genes was further upregulated in the LPS group, but not in other groups, by the short period of VILI indicating that these already inflamed lungs are more sensitive to the pro-inflammatory effects of injurious ventilation. NF- κβ was not upregulated and was unaffected by VILI which we attribute to the 48 h time interval between exposure and measurement. The LPS exposure produced broad swaths of atelectasis with numerous cell infiltrates that were typically located adjacent to the conducting airways (Figure 7). Stereological estimations characterized LPS injury with increased septal tissue volume compared to all other groups (Figure 8B), a modest amount of airspace edema (Figure 8D), and loss of gas-exchanging surface area (Figure 8E) that are in agreement with prior reports, e.g., (de Souza Xavier Costa et al., 2017). Interestingly, lung stiffness was only significantly affected at low PEEPs (Figure 1) and PV loop delivered volumes (Figure 4D) were no different than healthy controls. These mechanics are in harmony with the morphometry, where LPS parenchymal air volumes (Figure 8A) were decreased at low pressures and recovered to CTL level by *p* = 5 cmH_2_O. The mean linear intercept length (Figure 9A) trended upward at *p* = 5 and 10 cmH_2_O. Taken together, the mechanical and structural data suggest that the open parenchyma in the LPS group has relatively normal stiffness, and that airspaces are somewhat dilated at higher pressures to yield relatively large and compliant lungs.

In striking contrast to LPS is LAV, where there is no elevation in TNF-α, CXCL-1, IL-6, and NF- κβ immediately following the saline lavage and only a modest trend upward after a short period of VILI (Figure 11). BALF protein is unaffected prior to ventilation, and then trends upwards with 25 min of high-pressure VILI (Figure 10). These outcomes are not unexpected due to the short interval between lavage and measurement, the mild nature of the instillate, and the brief duration of VILI. Nevertheless, the changes in lung mechanical function are more pronounced in LAV than the other injury groups. PV loops show lower lung volumes, higher hysteresis area, and quasi-static compliance than all other groups (Figure 4) while H is significantly elevated at all PEEPs on the ascending ladder (Figure 1).

The LAV group is differentiated from the other injuries, particularly LPS, by alveolar instability (recruitment and derecruitment) that is shown in significant reductions in H at each PEEP when comparing the Descending to Ascending limbs of the PEEP ladder (Figure 1) as well as in the morphometric quantification of alveolar collapse (Figure 8C) and surface area (Figure 8E). The recruitment/derecruitment dynamics are further clarified in Figure 6 which shows H separated by both injury group and PEEP ladder ventilation pattern. In the LAV + VT6RMs group, with low tidal volume ventilation and recruitment maneuvers at each PEEP change, there is a drop in elastance at PEEP = 3 vs. 0 cmH_2_O on the Ascending ladder, indicating that inflation to 30 cmH_2_O (the RM) is sufficient to recruit collapsed units, and that PEEP = 3 cmH_2_O is sufficient to prevent some derecruitment. There is a further decrease in elastance at PEEP = 6 cmH_2_O in LAV + VT6RMs which, taken together with H on the descending ladder, suggest derecruitment occurs at airway pressures ≲ 6 cmH_2_O in that injury model. The morphometric analysis supports that assertion, showing a significant increase in the fraction of collapsed septal tissue as airway pressure is decreased from 10 to 5 cmH_2_O which is accompanied by a loss of gas-exchanging surface area (Figures 8C,E). This increased collapse and loss of surface area is likely due to the combination of derecruitment of whole alveoli (Pavone et al., 2007; Perlman et al., 2011; Tabuchi et al., 2016; Knudsen et al., 2018) and folding of the intra-alveolar septa (Tschumperlin and Margulies, 1999; Knudsen et al., 2018). Note that the tissue was fixed for morphometry after inflation to 30 cmH_2_O (an RM) and then deflation to the airway pressure where the analysis was performed.

To estimate LAV recruitment pressures, the mean peak inspiratory pressure ( $\bar{PIP}$ ) was calculated for each ventilation pattern at each PEEP on the Ascending ladder. There are no significant differences in H between VT6RMs and VT10 at PEEP = 9 cmH_2_O (Figure 6B) where $\bar{PIP}$ = 26.5 cmH_2_O for VT10, suggesting equal recruitment. Likewise, there is no significant difference in H between VT6 and all other groups at PEEP = 15 cmH_2_O where $\bar{PIP}$ = 26.7 cmH_2_O for VT6. From that data, it can be surmised that most recruitment in LAV occurs below ≈25 cmH_2_O, the pressure at which H for the VT6 and VT10 groups normalizes to VT6RMs. Further evidence for that upper limit of recruitment pressure comes from the absence of additional reductions in stiffness when comparing VT6 ( $\bar{PIP}$ = 26.7 cmH_2_O at PEEP = 15) to VT6RMs (RM PIP = 30 cmH_2_O at all PEEPs) and the VT10 ( $\bar{PIP}$ = 39.3 cmH_2_O at PEEP = 15).

In comparison to LAV, recruitability in the HCL injury group is more subtle. Figure 6D shows that H for HCL + VT6RMs and HCL + VT10 tend to be less than HCL + VT6, following a similar pattern to LAV. However, these differences are much less pronounced in HCL than LAV, indicating a smaller fraction of alveoli are recruitable as supported by the morphometry (Figure 8C). The common factor between HCL and LAV is increased H and a reduction in airspace size, particularly at an air inflation pressure *p* = 10 cmH_2_O (Figure 9A), where there is a left-shift of the intercept lengths (Figures 9B,C) that is indicative of smaller alveoli and suggests degradation of the pulmonary surfactant system. It is interesting to see that those two groups show shared signs of high surface tension (smaller airspaces) but do not exhibit similar recruitability, and this may be due to the 48 h duration of HCL injury vs. the ∼30 min duration of LAV injury. The HCL group was alone in demonstrating increased Newtonian resistance (Rn) which is indicative of damage to the airways. We did not seek to quantify airways morphometry, but qualitative observations indicate airway epithelial cell injury and edema (Figure 7D).

The differences in lung structure and function hold implications for mechanical ventilation strategies to reduce or prevent VILI while maintaining gas exchange. In the case of CTL and LPS there is little recruitability and, in LPS, airspace enlargement is observed at higher pressures. Ventilation at lower PEEPs will reduce static septal strain while providing sufficient aerated septa for gas exchange (Figure 8C), although this may be less of a concern since elevated static strains are suggested to be less damaging than high dynamic strains (Protti et al., 2014). In contrast, the high recruitability of the LAV group, and to a lesser degree HCL, that is demonstrated in elastance (Figure 1) and morphometry (Figure 8C) suggests that recruitment, either with high PEEPs or RMs, followed by ventilation with higher PEEPs, will improve gas exchange by increasing septal surface area (Figure 8E) and reduce driving pressures leading to less VILI (Aoyama et al., 2018). Further study is necessary to identify how these injury etiologies interact with PEEP to affect VILI pathogenesis, and such analyses may help target interventions and trials to improve ARDS outcomes (Goligher et al., 2015).

This investigation provides a detailed assessment of the effects of lavage, acid, and endotoxin-induced lung injury on lung structure, function, inflammation, and alveolocapillary barrier integrity. Nevertheless, there are several important limitations that should be noted. First, the use of mice eliminates the gravitational gradient that is an important contributor to mechanical function and VILI (Plataki and Hubmayr, 2010). The choice of species also affects the immune response (Matute-Bello et al., 2008). This is particularly important in LPS, and to a lesser degree HCL, where the 2 days of exposure provide time for the inflammatory response to affect structure and function. Second, we induced VILI with 25 min of high pressure, zero PEEP ventilation which may cause differential effects when compared to days or weeks of clinical low tidal volume ventilation. Finally, these mouse ALI models do not fully capture the full scope of clinical ARDS which may include multiple contributing factors and, potentially, includes a fibroproliferative phase.

In summary, we measured the effects of lavage (LAV), endotoxin (LPS), and hydrochloric acid (HCL) injury with a 2^nd^ hit of ventilator-induced lung injury alongside healthy controls. The LAV group showed the most dramatic increases in lung elastance, which was moderated by recruitment, and airspace size was reduced. The short period of VILI induced alveolar leak but not significant inflammation. The LPS group showed only modest increases in elastance at lower levels of inflation and did not demonstrate significant recruitability. Instead, that injury was characterized by inflammation, which was exacerbated by ventilation, along with widespread atelectasis and septal swelling. The HCL group had increased elastance and showed a modest degree of recruitability. Pro-inflammatory gene expression was somewhat elevated and airspace dimensions were markedly reduced at higher inflation pressures. Taken together, these differential outcomes indicate that lung protective ventilation will vary as a function of injury etiology.

## Data Availability

The raw data supporting the conclusion of this article will be made available by the authors, without undue reservation.
