# Supplementary material for: Differential effects of two-hit models of acute and ventilator-induced lung injury on lung structure, function, and inflammation
Source: Front Physiol. 2023 Jul 26;14:1217183. doi: 10.3389/fphys.2023.1217183 (PMC10410077; doi:10.3389/fphys.2023.1217183)
Supplement: Supplementary file 1 [file DataSheet1.PDF]

Supplementary Material

1 Supplementary Figures

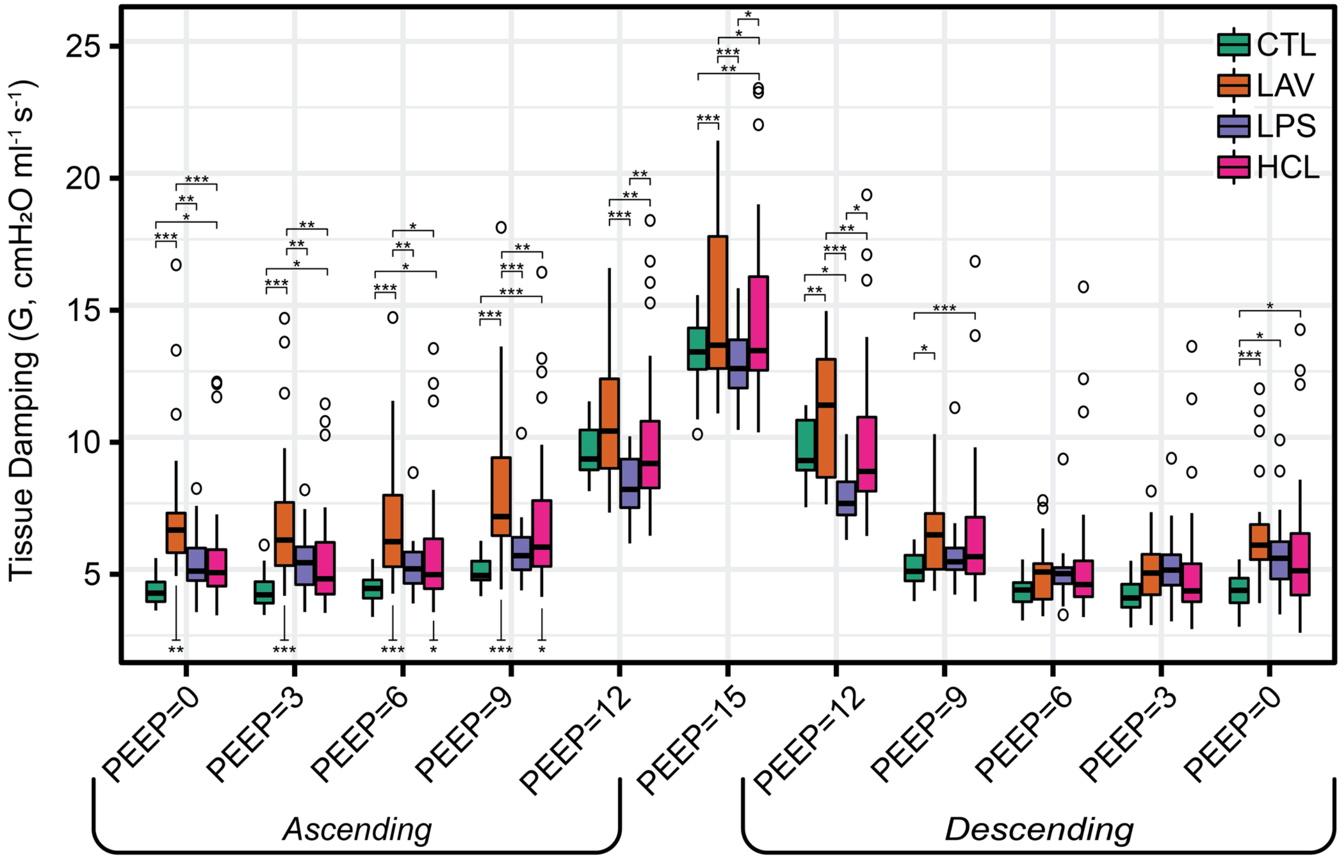

Supplementary Figure 1. Tissue damping (G) measured during the PEEP ladders with PEEP incrementing from 0 to 15 cmH_2O (Ascending) and then decreasing from 15 to 0 cmH_2O (Descending). Symbols above the bars indicate significant inter-group difference at that PEEP; symbols below bars indicate significant intra-group differences from Ascending to Descending.

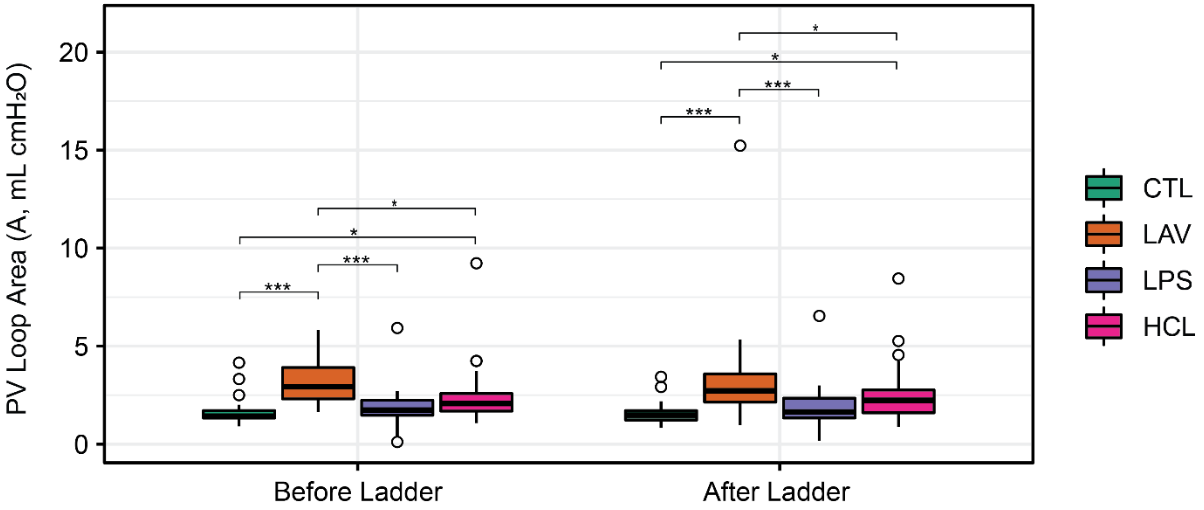

Supplementary Figure 2: Quasi-static pressure-volume loop areas recorded before and after the PEEP ladder are consistent within groups.

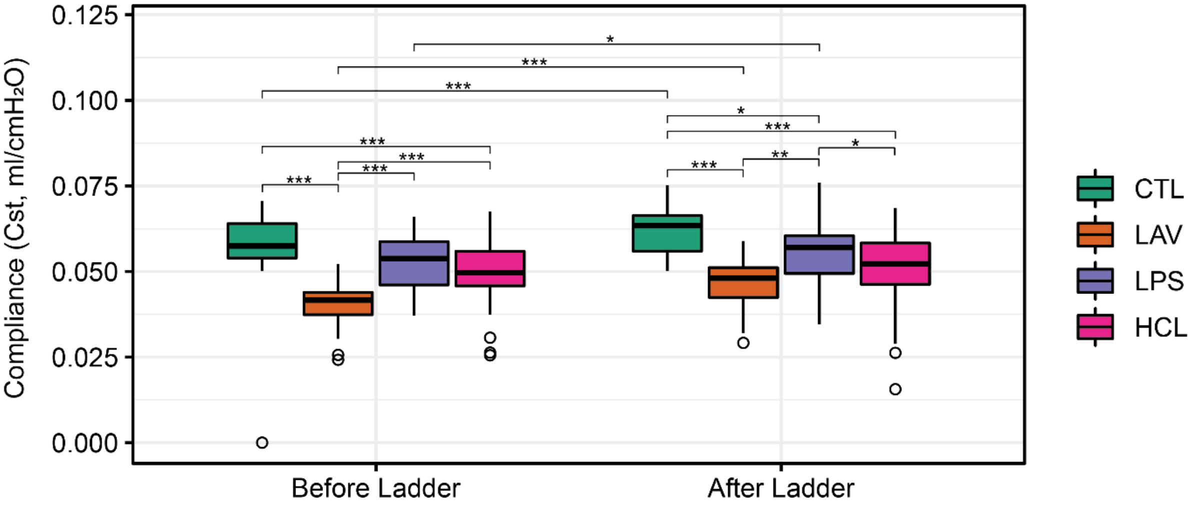

Supplementary Figure 3: Quasi-static compliance calculated at 5 cmH_2O on the descending limb of pressure-volume loops recorded before and after the PEEP ladders shows a modest but significant increase with ventilation in the CTL, LAV, and LPS groups.

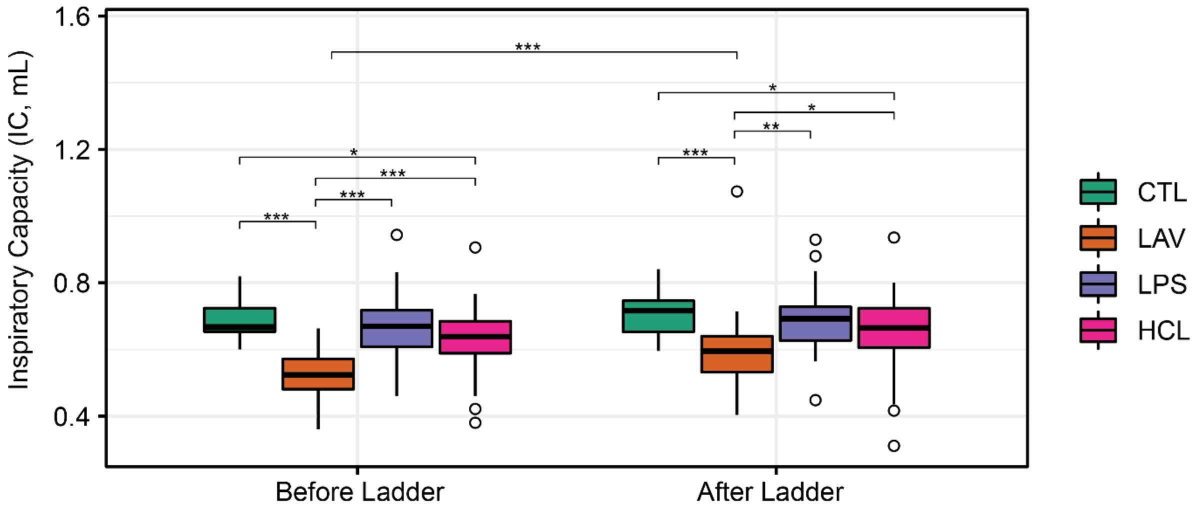

Supplementary Figure 4: Inspiratory capacity, the volume delivered in the quasi-static pressure volume loops, before and after the PEEP ladders is significantly increased with ventilation in LAV.

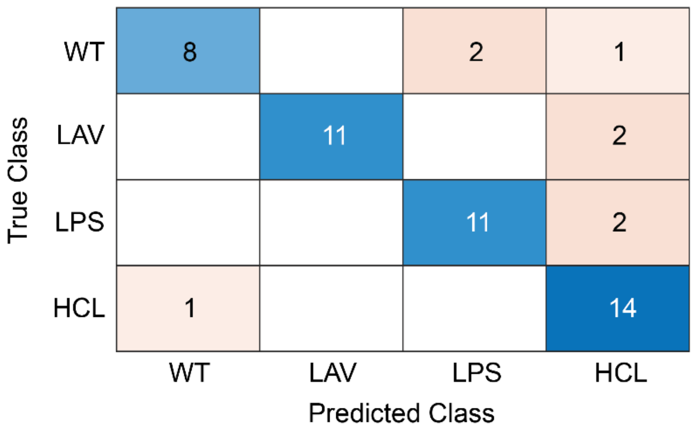

Supplementary Figure 5: Confusion matrix demonstrating the predictive accuracy of the multinomial ridge regression model with the true (known) experimental group on the vertical axis and the model-predicted experimental group on the horizontal axis.

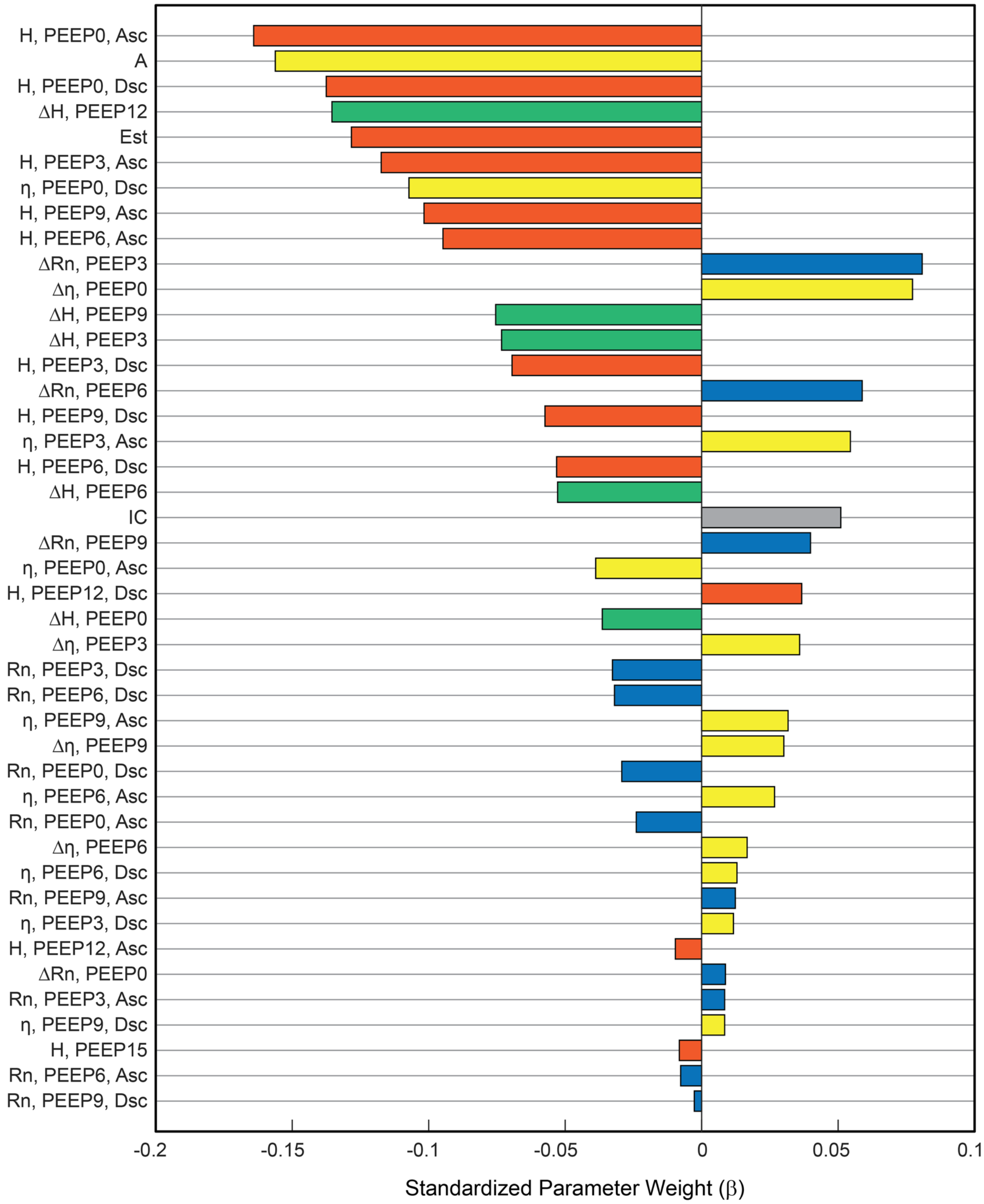

Supplementary Figure 6: Multinomial ridge regression for the control (CTL) group showing the normalized regression parameters rank-ordered by magnitude. Large positive values indicate that high values for that parameter are an important classifier for the CTL group; large negative values indicate a strong inverse relationship. Colors indicate parameters related to stiffness (orange), recruitment (ΔH, green), Newtonian resistance (blue), hysteresivity and PV loop area (yellow), and PV loop delivered volume (grey). Parameters from the PEEP ladders are described by the parameter name (H, Rn, or η), the measurement PEEP, and if the measurement was recorded on the Ascending (Asc) or Descending (Dsc) limb of the PEEP ladder.

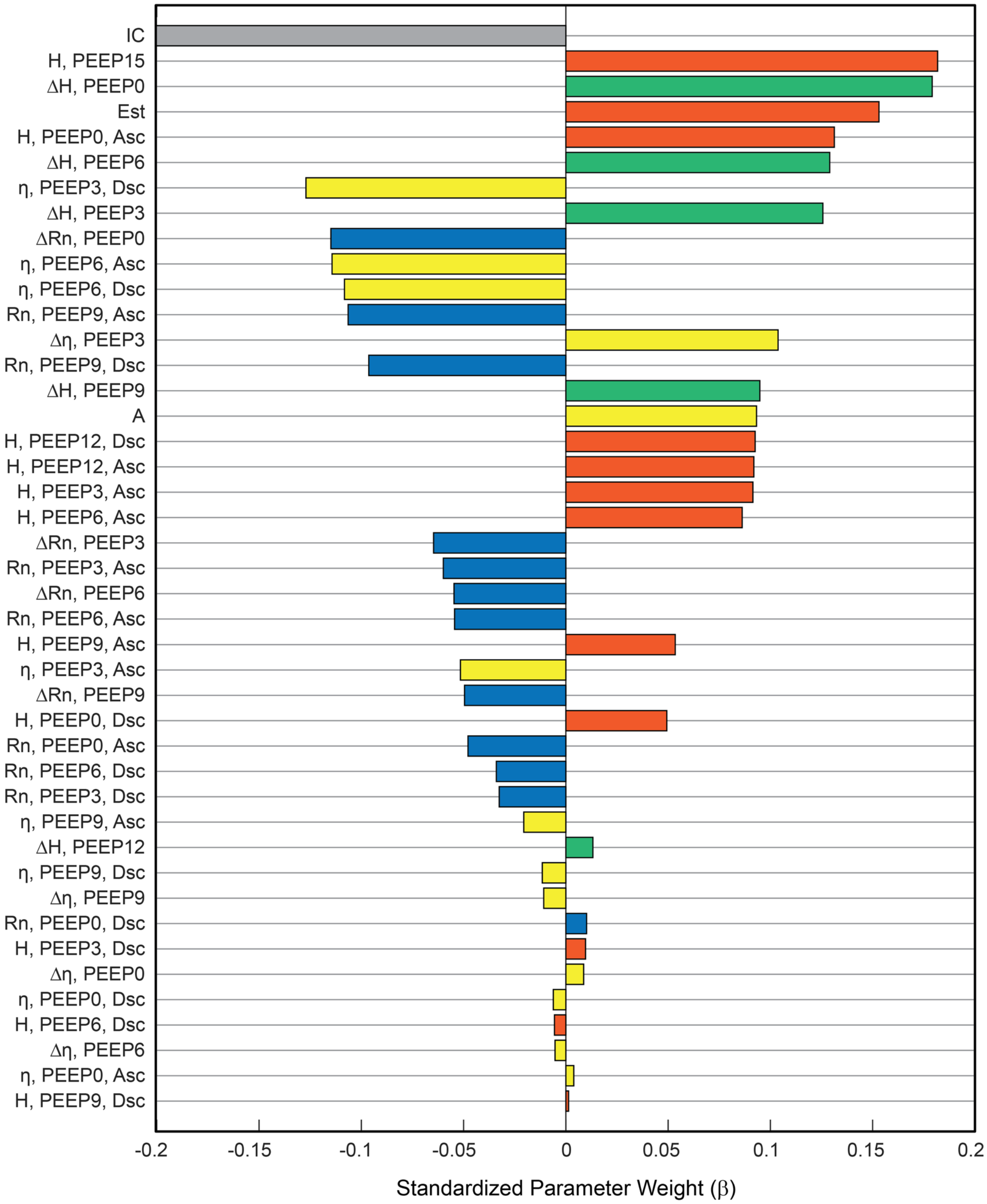

Supplementary Figure 7: Multinomial ridge regression for the lavage (LAV) group showing the normalized regression parameters rank-ordered by magnitude. Large positive values indicate that high values for that parameter are an important classifier for the LAV group; large negative values indicate a strong inverse relationship. Colors indicate parameters related to stiffness (orange), recruitment (ΔH, green), Newtonian resistance (blue), hysteresivity and PV loop area (yellow), and PV loop delivered volume (grey). Parameters from the PEEP ladders are described by the parameter name (H, Rn, or η), the measurement PEEP, and if the measurement was recorded on the Ascending (Asc) or Descending (Dsc) limb of the PEEP ladder.

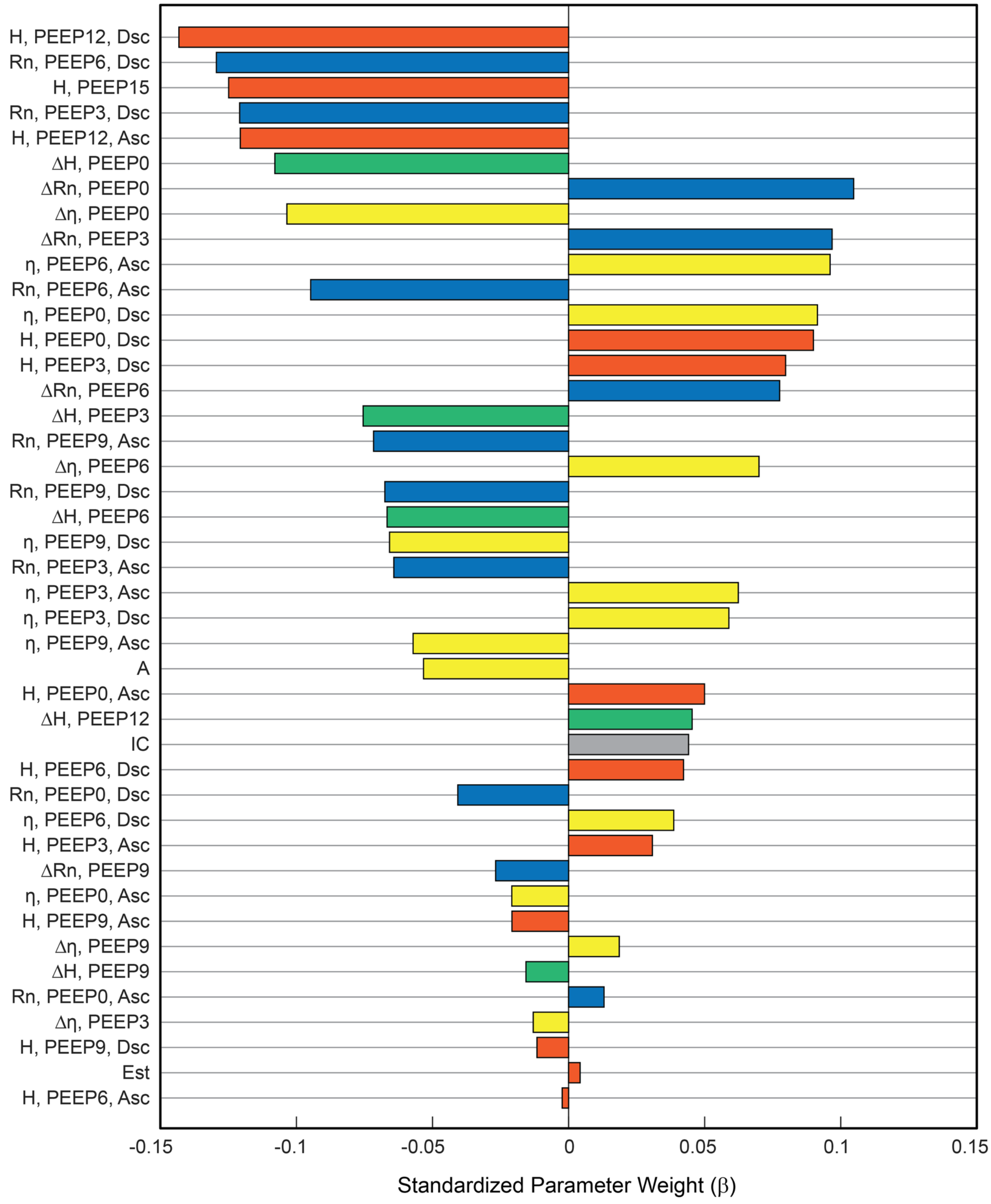

Supplementary Figure 8: Multinomial ridge regression for the endotoxin (LPS) group showing the normalized regression parameters rank-ordered by magnitude. Large positive values indicate that high values for that parameter are an important classifier for the LPS group; large negative values indicate a strong inverse relationship. Colors indicate parameters related to stiffness (orange), recruitment (ΔH, green), Newtonian resistance (blue), hysteresivity and PV loop area (yellow), and PV loop delivered volume (grey). Parameters from the PEEP ladders are described by the parameter name (H, Rn, or η), the measurement PEEP, and if the measurement was recorded on the Ascending (Asc) or Descending (Dsc) limb of the PEEP ladder.

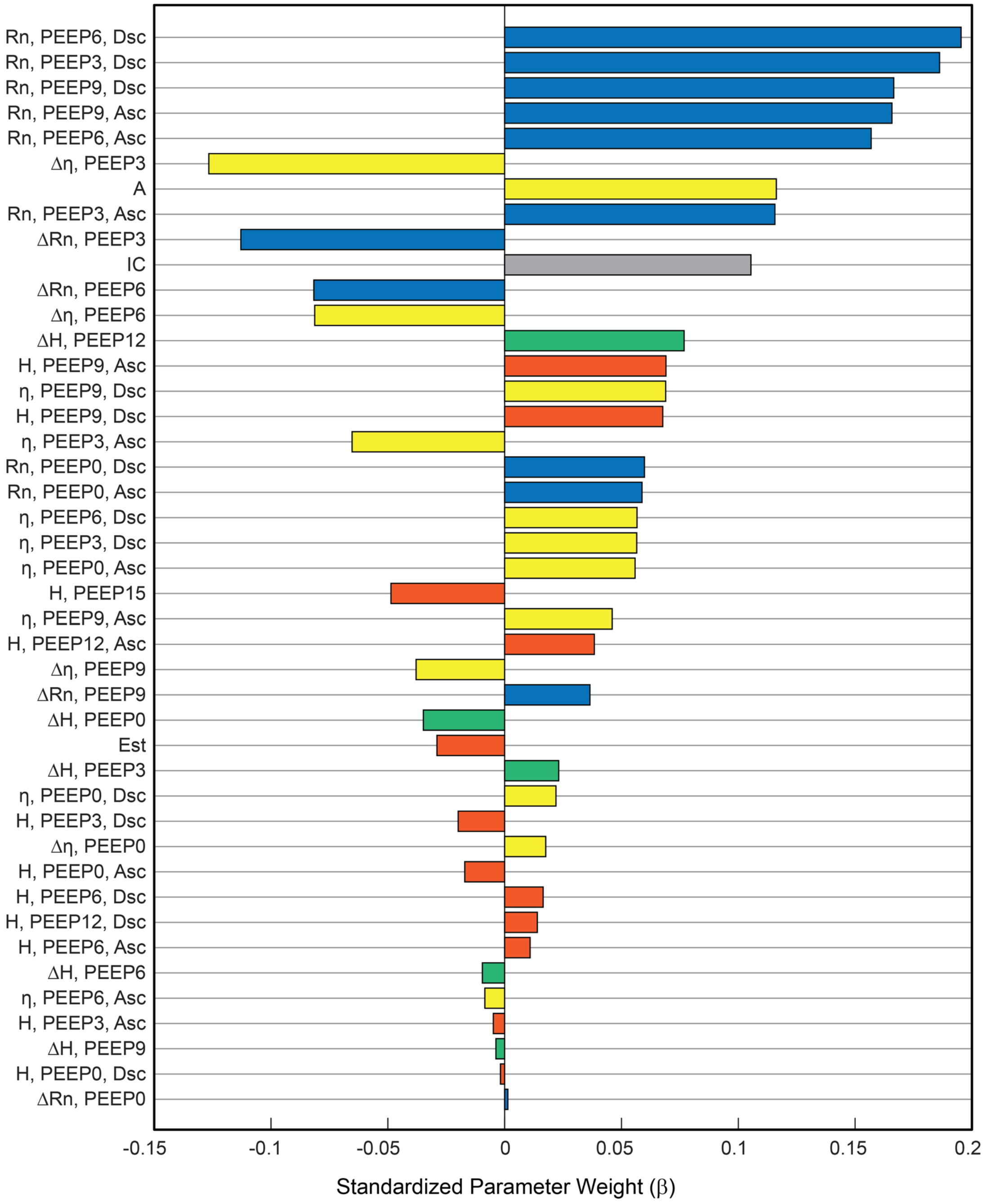

Supplementary Figure 9: Multinomial ridge regression for the acid injury (HCL) group showing the normalized regression parameters rank-ordered by magnitude. Large positive values indicate that high values for that parameter are an important classifier for the HCL group; large negative values indicate a strong inverse relationship. Colors indicate parameters related to stiffness (orange), recruitment (ΔH, green), Newtonian resistance (blue), hysteresivity and PV loop area (yellow), and PV loop delivered volume (grey). Parameters from the PEEP ladders are described by the parameter name (H, Rn, or η), the measurement PEEP, and if the measurement was recorded on the Ascending (Asc) or Descending (Dsc) limb of the PEEP ladder.

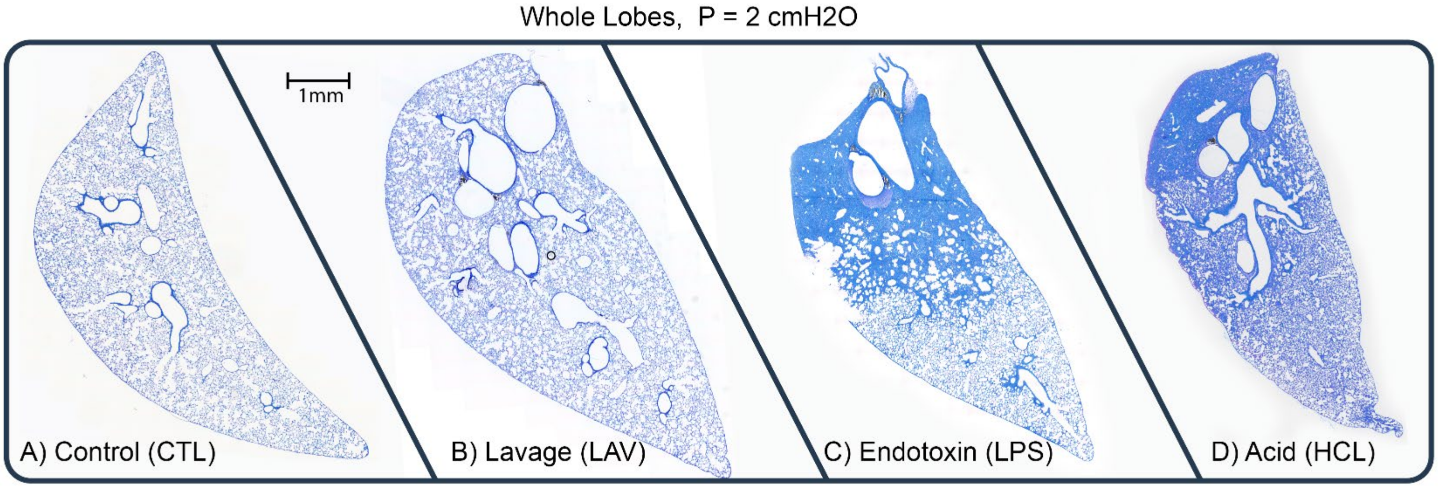

Supplementary Figure 10: Representative micrographs showing whole lobes of perfusion-fixed lung tissue that were air-inflated to P = 2 cmH_2O and stained with Toluidine blue. The control (CTL), lavage (LAV), endotoxin (LPS), and acid injury (HCL) groups are shown in the 1st through 4th columns.

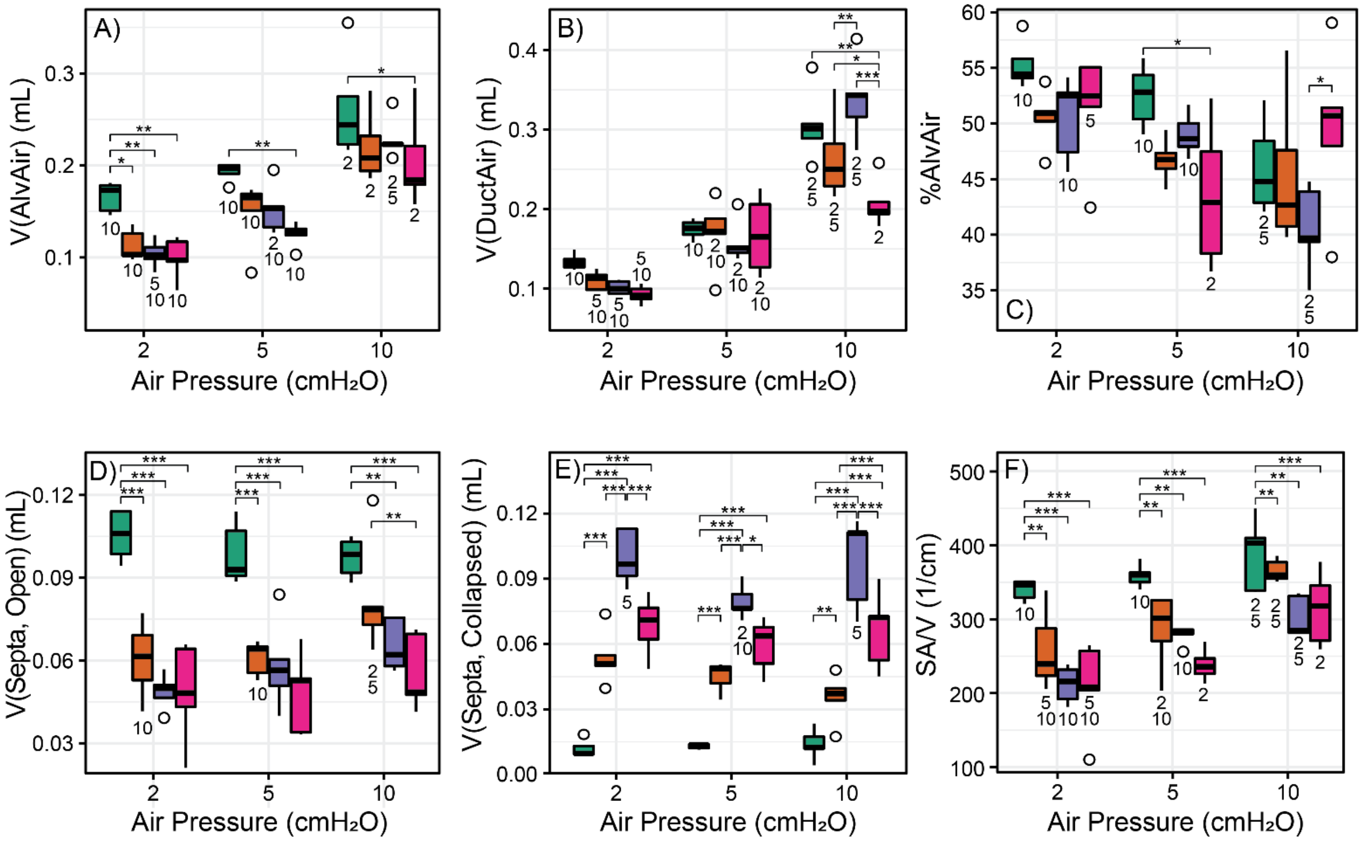

Supplementary Figure 11: Lung morphometry (stereology) performed at air inflation pressures of P = 2, 5, and 10 cmH_2O (horizontal axes); the four experiment groups are shown in green (CTL), orange (LAV), lavender (LAV), and magenta (HCL). The volume of alveolar air (V(AlvAir), A) is reduced in all injury groups at P=2 and in HCL at P=5 and 10 cmH_2O. The volume of air in the alveolar ducts (V(DuctAir), B) is reduced at P=10 in the LAV and HCL groups. There is a trend towards shifting ventilation to the alveolar ducts with injury (C) except for at P=10 in HCL. The volume of aerated septa (V(Septa, Open), D) is higher in CTL than all injury groups at all pressures. The volume of collapsed septa (V(Septa, Collapsed), E) is low in CTL and higher in LPS than all other groups. The parenchymal surface area to volume ratio (SA/V, F) is higher in CTL than all other groups and, in all groups, significantly increases with increasing pressure. Significant differences at each pressure are indicated with symbols above the bars; significant differences between pressures for each group are indicated with number below the bars (the number indicates the pressure that data is different from).

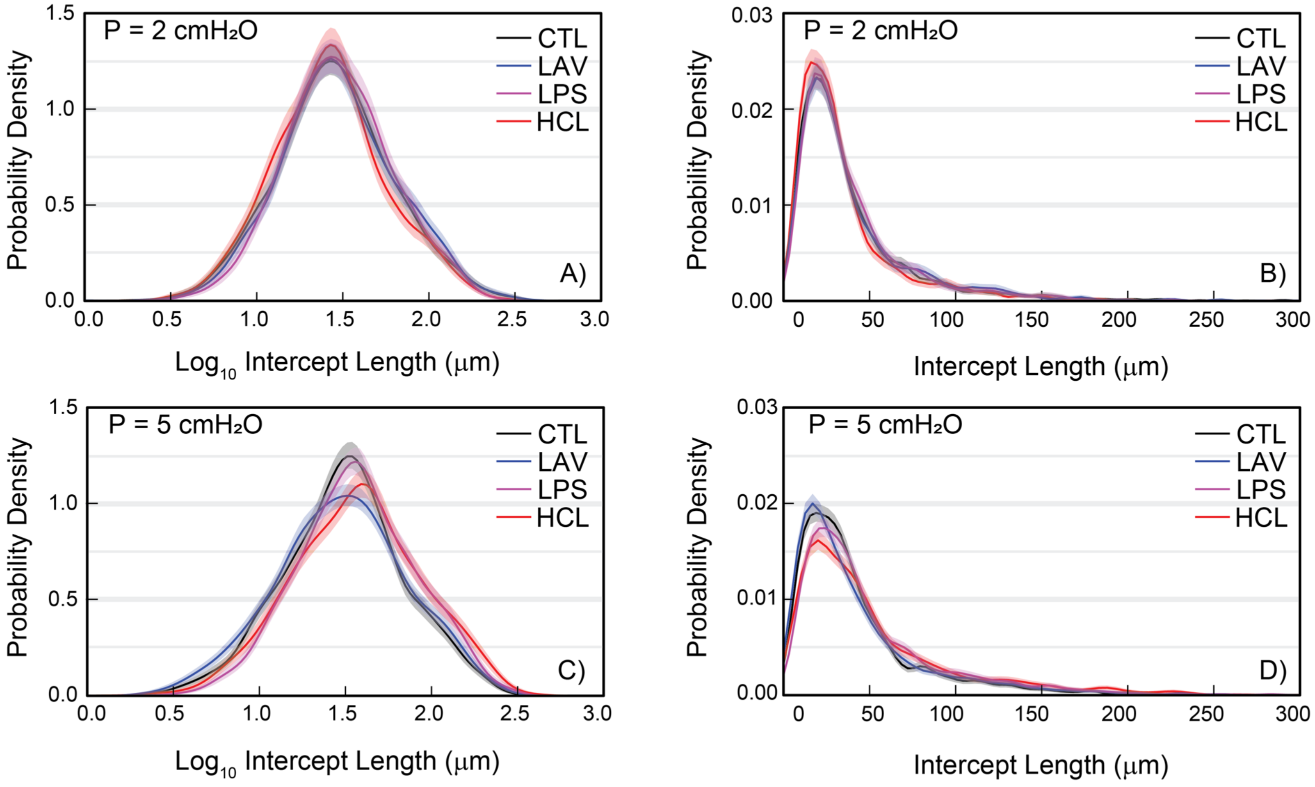

Supplementary Figure 12: Average probability density functions for intercept length at pressures of 2 (A) and 5 (B) cmH_2O. Panels (C) and (D) show the log_{10} intercept length for each experimental group.
